# Maize (*Zea mays* L.) survival on Mars depends on regolith’s chemical composition rather than reduced gravity or lack of magnetic field

**DOI:** 10.1038/s41598-026-50840-4

**Published:** 2026-04-28

**Authors:** Giulia Pia Servetto, Paolo Botta, Gioele Rossi, Andrea Bernasconi, Francesco Caldo, Ruggero Vigliaturo, Massimo E. Maffei

**Affiliations:** 1 Department of Earth Sciences, University of Turin, Turin, Italy; 2 Plant Physiology Unit, Department of Life Sciences and Systems Biology, University of Turin, Turin, Italy; 3 Interdepartmental Centre for Studies on Asbestos and Other Toxic Particulates “G. Scansetti”, University of Turin, Turin, Italy

**Keywords:** Chemical characterization, Martian regolith simulant, Simulated reduced gravity, Random positioning machine, Hypomagnetic field, PIN genes, Redox homeostasis, Biochemistry, Plant sciences

## Abstract

The long-term success of human missions to Mars requires the ability of plants, like maize (*Zea mays* L.), to thrive under Martian conditions. We assessed the combined effects of the planet’s unique biophysical environment by simulating Martian gravity (sMG, 0.38 g) and Mars hypomagnetic field (hMF, ~ 40 nT) and the chemical toxicity of Mars Global Simulant (MGS-1) regolith. We conducted comprehensive mineralogical characterization of MGS-1 using PXRD, SEM-EDXS, and S/TEM-EDXS. Maize was cultivated in a 30% MGS-1 substrate under sMG and hMF conditions. Analyses included morphological assessments, biochemical measurements, and gene expression. MGS-1 characterization revealed mineral phases (e.g., quartz, mica), enhancing its chemical complexity. Applied alone, hMF and sMG triggered specific compensatory responses in auxin transport and metabolism (e.g., sMG increased leaf area; hMF modulated protein content). In dual-stress scenarios, MGS-1 emerged as the dominant stressor, overriding biophysical cues. The hMF + MGS-1 combination enabled metabolic retention (elevated protein and root *PIN* plasticity), indicating manageable stress. Conversely, sMG + MGS-1 posed the greatest threat, inducing systemic metabolic depletion (decreased protein and proline) and disrupting root development signals (*PIN* downregulation). Ultimately, maize survival on Mars is primarily constrained by regolith chemical imbalance and toxicity, rather than reduced gravity or hypomagnetism alone.

**Supplementary Information:**

The online version contains supplementary material available at 10.1038/s41598-026-50840-4.

## Introduction

Life on Earth has evolved under specific abiotic conditions, making extraterrestrial environments hostile to terrestrial organisms^1^. For plants specifically, the extreme extraterrestrial environments, characterized by low gravity, radiation, and pressure and temperature extremes, present significant challenges to growth on other planets^2^. Mars exemplifies this harshness: its gravity is approximately 38% of Earth’s (with *g* = 3.71 m s^−2^ compared to Earth’s *g* = 9.81 m s^−2^), it lacks a magnetosphere, resulting in a surface ionizing radiation of about 30 µSv h^−1^ (versus Earth’s 0.1–0.2 µSv h^−1^), and its atmospheric pressure is about 1% of Earth’s. Under these radically different conditions, plants would encounter environments vastly dissimilar to those in which they evolved. Therefore, among the various stressors in the Martian environment, on-Earth experiments can simulate key factors such as regolith composition, reduced gravity, and hypomagnetic fields to investigate the feasibility of plant cultivation in extraterrestrial outposts like Mars.

The surface of Mars is covered by a loose, unconsolidated material called regolith. The mineralogical evolution of this material is determined by its primary mineralogy and complex interplay of processes, including impact comminution, mechanical erosion by wind, water, and lava, chemical weathering by fluids and oxidants, and meteoritic impacts^3,4^. To facilitate research, regolith simulants such as the Mars Global Simulant 1 (MGS-1), the Mojave Mars Simulant (MMS), the new Martian soil simulant created using MMS base (JSC-RN), just to list a few, have been developed to closely mimic the Martian regolith’s composition and physical properties^5,6^. These regolith simulants offer a controlled laboratory setting to investigate plant growth responses, evidence limiting factors, and elaborate strategies for optimizing plant performance^7^. Using regolith simulants provides a vital test bed for studies exploring the potential of Mars’ regolith and bedrock in agricultural applications. They help in understanding data gaps of these materials, which will require further characterization, and in developing methods to conduct the necessary in-situ characterization of true Martian regolith and bedrock^8^. By studying plant growth using simulated substrates, researchers can gain valuable insights into the challenges and opportunities associated with extraterrestrial agriculture (or space farming)^9^.

Various plant-based studies have already utilized Mars simulants^10^. The MGS-1 standard is one such simulant, modelled on the wind-blown soil of Rocknest in Gale Crater, representing average basaltic regolith found on Mars^11^. However, studies have confirmed the absence of loosely bound water and its hydrophobic nature, significantly hindering plant growth^4^. In pure form, MGS-1’s physico-chemical properties preclude plant development. For instance, crops like wheat (*Triticum aestivum*), oat (*Avena sativa*), alfalfa (*Medicago sativa*), mung bean (*Vigna radiata*), garden cress (*Lepidium sativum*), and white mustard (*Sinapsis alba*) died after only six days of growth in pure MGS-1^4^. Similar inhibition occurred in sweet potato (*Ipomoea batata*)^12^. Lettuce (*Lactuca sativa* var. *longifolia*) and *Arabidopsis thaliana* successfully germinated in other Martian regolith simulants (JSC-Mars-1 A and MMS-1) but growth was inhibited, with seedlings dying nine days post-germination without nutrients^13^. Thus, mixing MGS-1 with water-retaining substrates is essential for initial growth. While some plants grow on simulants without added nutrients, organic matter, fertilizers or symbiotic interactions markedly improve growth and yield^14,15^. Growing *A. thaliana* on Apollo lunar regolith showed metabolic and morphological stress, exhibiting responses characteristic of salt stress and reactive oxygen species (ROS) generation^16^. This aligns with Moon regolith simulants, where oxidative stress responses predominate^17^.

Reduced gravity induces metabolic changes in plants, altering cellulose, hemicelluloses, lignin, and callose content^18^. It disrupts subcellular organization, including chloroplasts, mitochondria, endoplasmic reticulum, and cytoskeleton^19,20^. Cell cycle, meristematic activity, and nucleolar function are also impacted^21,22^. Thus, Martian gravity (0.38 g) is expected to cause significant shifts. Random Positioning Machines (RPMs) provide a valuable methodology for simulating micro- and low-gravity conditions^23,24^ by continuously altering gravitational direction, minimizing unilateral effects without reducing magnitude^25^. Reduced gravity minimally affects seed germination but influences orientation-dependent growth, making RPMs ideal for altered gravity studies^26^. For example, under simulated Moon gravity, Arabidopsis showed increased cell proliferation but depleted growth^27^.

Altering the Geomagnetic Field (GMF), through both hypomagnetic (hMF) and hypermagnetic (HMF) conditions induces responses in model and crop plants^28^. Under reduced gravity, high gradient magnetic fields (HGMF) are capable of inducing curvature in roots and shoots through the displacement of amyloplasts^29^. Conversely, reducing the GMF to a near-null (like the conditions on Mars) values elicits plant abiotic stress responses^30^, including decreased ROS production^31^, reduced photosynthetic efficiency^32^, altered flowering^33^, modified pathogen responses^34,35^, and photoreceptor changes^36,37^. Intriguingly, plants possess homologs of the *Drosophila melanogaster* magnetoreceptor (MagR)^38^, which are modulated by hMF^39^.

Maize (*Zea mays* L.) is a globally vital crop, providing food, feed, and raw materials with essential nutrients like carbohydrates, proteins, vitamins, and minerals, especially in developing countries^40^. To our knowledge, literature regarding the response of maize to Mars regolith simulant is missing.

The goal of this work was to assess maize responses to simulated Martian conditions: regolith, simulated reduced gravity (simulated fractional gravity (0.38)), and hypomagnetic field. Despite numerous Mars simulation facilities^41^, no data currently exist on combining Mars regolith with Mars simulated gravity or magnetic fields. Here, we characterize MGS-1 and show that its use with RPM-simulated Mars gravity (0.38 g), and hMF (~ 40 nT with a triaxial Helmholtz coils system) induces specific stress responses in the early maize development.

## Results

The following results first detail the mineralogical characterization of the MGS-1 regolith simulant using advanced analytical techniques, which directly informs the subsequent biological assays by revealing chemical factors (e.g., mineral dissolution and nutrient bioavailability) that underpin maize’s adaptive responses to Martian stressors. Mineralogical analyses, including Powder X-ray Diffraction (PXRD), Scanning Electron Microscopy-Energy Dispersive X-ray Spectroscopy (SEM-EDXS), and Scanning/Transmission Electron Microscopy-EDXS (S/TEM-EDXS), revealed the complex phase composition of MGS-1, confirming major components and identifying undeclared minerals phases by the manufacturer’s compositional data (see Supplementary material, Table S1, item 3.3 “mixture”).

### PXRD analyses shows dominant plagioclase along with trace minerals

**Fig. 1:**
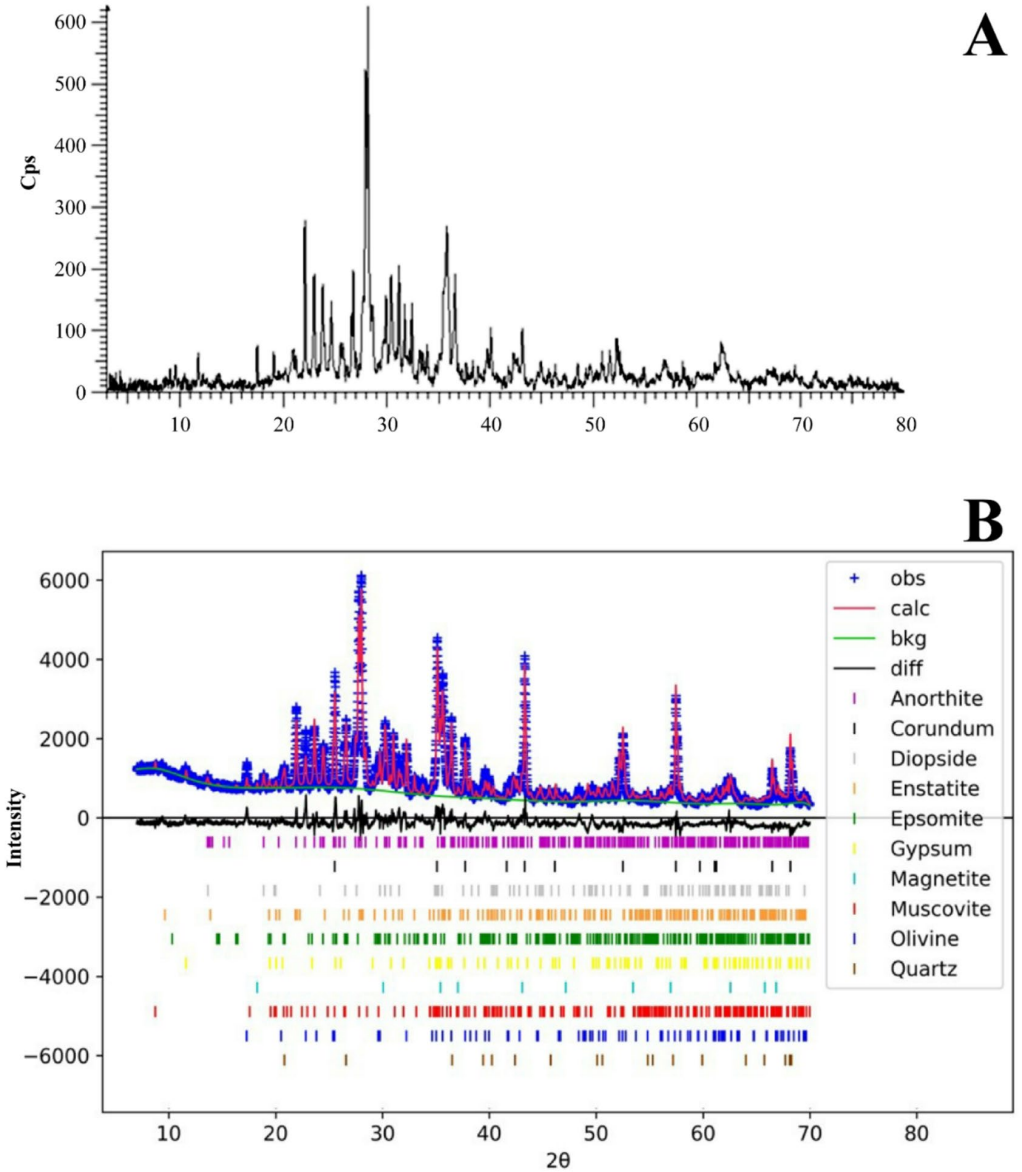
Plagioclase (anorthite, CaAl2Si2O8) is the dominant crystalline mineral phase. (**A**) PXRD spectrum of MGS-1, and (**B**) Rietveld refinement graphical fit by GSAS-II software.

Plagioclase (anorthite, CaAl_2_Si_2_O_8_) was identified as the dominant crystalline mineral phase (Fig. 1A and B). The presence of olivine and pyroxenes was also confirmed by the PXRD analysis. In the case of gypsum (CaSO_4_), magnetite (Fe_3_O_4_), and epsomite (Mg[SO₄] · 7 H₂O), although their main peaks were detected, these peaks showed very low counts per second (cps), suggesting these phases are present only in trace amounts. Furthermore, peaks corresponding to both siderite (FeCO₃) and hematite (Fe₂O₃) were not found. Furthermore, the PXRD spectra revealed the presence of quartz (SiO_2_) and mica phases, which were not listed in the compositional datasheet provided by the company. The graphical fit obtained from the Rietveld refinement is displayed in Fig. 1B. The refinement achieved a reliable weighted-profile R_WP_ factor of 10.8%.

### SEM-EDXS analyses confirm mineral phases and reveal the presence of diatom

**Fig. 2:**
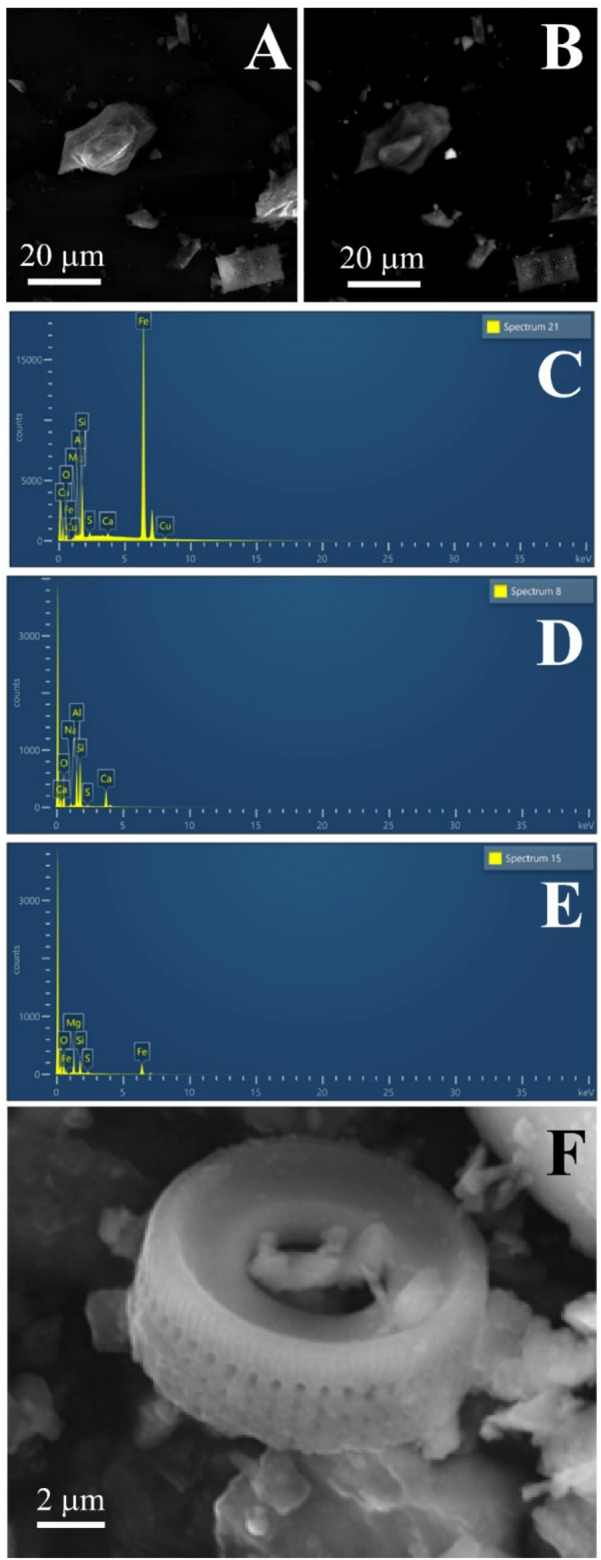
SEM images in secondary electrons (**A**) and backscattered electrons (**B**) of the MGS-1. EDXS spectra of a possible pyroxene (**C**), anorthite (**D**) and olivine (**E**) particles detected in the MGS-1 sample. (**F**) SEM image example of a diatom present in MGS-1.

The SEM-EDXS chemical analyses performed on the MGS-1 confirmed the mineral phases previously identified by PXRD (Fig. 2A, B). Specifically, the collected EDXS spectra showed chemical compositions that can be related to pyroxenes (Fig. 2C), anorthite (Fig. 2D), and olivine (Fig. 2E). It highlighted the presence of low levels of S in most of the analyzed spectra. Furthermore, the analysis revealed the presence of different species of diatoms (Fig. 2F) within the MGS-1^11^.

### S/TEM-EDXS analyses reveal nanoscale mineral phases including aenigmatite, pargasite, and augite with widespread sulfur detection

S/TEM-EDXS HR analyses were performed on several particles in the MGS-1. The mineral phases identified are aenigmatite, pargasite, augite, hematite, and fayalite, quartz and mica (Table 1). In contrast to the bulk PXRD analysis, no plagioclase phases were identified at this scale. As highlighted by SEM-EDXS investigation, the widespread presence of S was also detected in the S/TEM-EDXS spectra collected on the MGS-1 particles.

**Table 1:** List of particles observed in MSG-1 by S/TEM-EDXS investigations.

Particle	Mineral phase identified	Chemical formula	Mineral group
1	Pargasite	NaCa_2_(Mg_4_Al)(Si_6_Al_2_)O_22_(OH)_2_	Amphibole
2	Aenigmatite	Na_4(_Fe^2+^_10_Ti_2)_O_4_[Si_12_O_36_]	Inosilicate
3A	Augite	(Ca, Na)(Mg, Fe, Al, Ti)(Si, Al)_2_O_6_	Pyroxene
3B	Hematite	Fe_2_O_3_	Metal oxide
3C	N.D	–	–
4	N.D	–	–
5A	N.D	–	–
5B	N.D	–	–
6A	N.D	–	–
6B	Fayalite	Fe_2_SiO_4_	Olivine

Two of the investigated particles were identified as inosilicates. The first particle (1 in Table 1) showed an irregular habit, and it was identified as pargasite, a complex amphibole inosilicate (R050321). The d-spacing (d_hkl_) measured on the IFFT image (Fig. S1) was compatible with the (0 4 4) plane of pargasite. The second inosilicate particle (2 in Table 1) had an irregular habit, and it was identified as aenigmatite (R061088). The d_hkl_ measured on the IFFT image (Fig. S2) were compatible with the (− 5 1 3) and (4 − 2 1) planes of aenigmatite.

The three particles identified in an aggregate were (Fig. S3): a particle (3A) having a polygonal morphology, and d_hkl_ compatible with augite (R061086); a particle (3B) having an irregular morphology, and d_hkl_ compatible with that of hematite (R040024) (Fig. S1C); The last particle (3C) presented an elongate morphology, and accordingly to the IFFT was amorphous, and containing O, Mg, Al, Si, Cr, and Fe.

Another isolated particle 4 exhibited a polyhedral morphology. The chemical EDXS analysis of this particle detected the presence of O, Na, Mg, Si, K, Ca, Ti, and Fe (Fig. S4), while its d_hkl_ were of 4.8 Å and 3.99 Å .

The aggregate 5 (Fig. S5) having a lamellar morphology composed by different particles with irregular shapes, including a particle (5 A) composed of O, Al, Si, and Fe and having d_hkl_ values of 3.19 Å and 3.15 Å. The chemical composition of particle 5B included O, Na, Mg, Al, Si, and Fe and d_hkl_ of 3.33 Å.

The last aggregate (6—Fig. S6) included two particles: a particle (6A) was composed of Mg, Al, Si, K, Ti, and Fe, whereas Particle 6B was identified as fayalite (an olivine group mineral, R070157), since the measured d_hkl_ was compatible with the (1 3 3) plane of fayalite.

The identified mineral species are compatible with the MGS-1 composition, and between the PXRD and SEM-EDXS investigation (Table 2). The slight deviation from the expected composition highlighted by the S/TEM-EDXS investigation is related to its poor sampling and the low number of investigated particles.

**Table 2:** Mineral phases identified in sample MSG-1.

MSG-1 SDS	PXRD	wt%	SEM-EDXS	S/TEM-EDXS
Plagioclase	Anorthite	30.3	Anorthite	Pargasite
Basaltic glass	Amorphous	43.2	Pyroxene	Aenigmatite
Pyroxene	Pyroxenes	7.7 (enstatite) 1.5 (augite)		Fayalite
Olivine	Olivine	10.5		Hematite
Epsomite	Epsomite	1.2		Amorphous
Ferrhydrite	Magnetite	1.5		
Hydrated silica	Gypsum	0.7		
Magnetite	Quartz	1.4		
Anhydrite/Gypsum	Mica	2.0		
Siderite				
Hematite				

This detailed mineralogical profile highlights potential sources of chemical stress, such as ion imbalances from pyroxene and olivine dissolution, which we hypothesize will dominate maize’s physiological and gene expression outcomes in the integrated biotic assays below.

### MGS-1 combined with agar reduces maize growth, development and ROS production

Preliminary experiments demonstrated that maize cannot survive in pure MGS-1 (data not shown). To enable plant survival and development, we tested increasing concentrations of an agar-amended substrate. We determined that a 30% MGS-1 concentration was suitable for plant germination, survival, and subsequent development. This concentration was therefore chosen for all experiments simulating Mars gravity and magnetic field conditions.

**Fig. 3:**
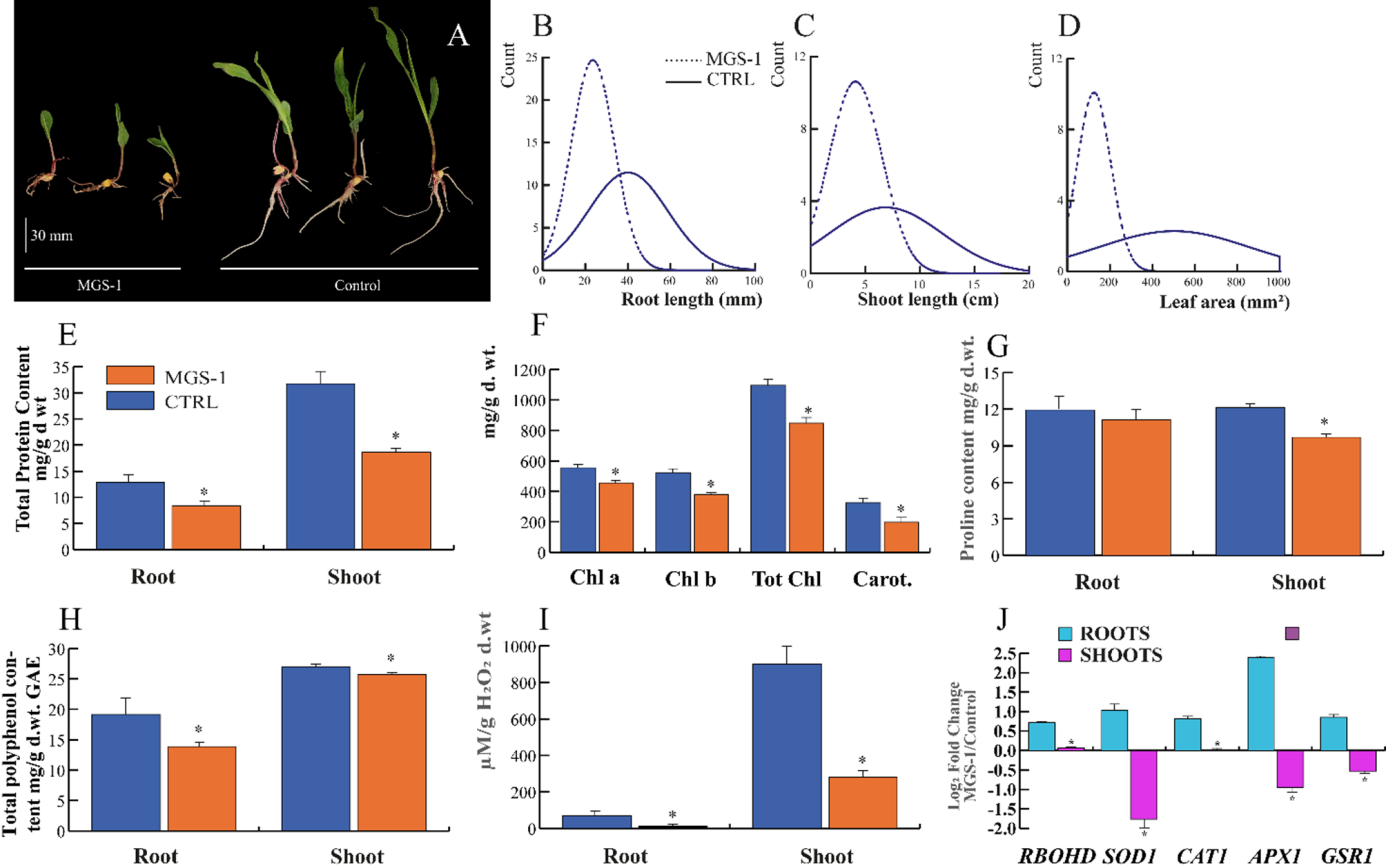
Morphological and biochemical changes in *Zea mais* grown on 30% MGS-1 and 70% half strength MS and Agar as compared to plants grown in the same condition without MGS-1 (CTRL). (**A**) Representative plants of the same age grown in MGS-1 and control conditions. (**B**) Density plot of root length. (**C**) density plot of shoot length. (**D**) Density plot of leaf area. (**E**) Total protein content. (**F**) Chlorophyll and carotenoid content. (**G**) Proline content. (**H**) Total polyphenol content. (**I**) steady-state production of H_2_O_2_. (**J**) Expression of genes involved in ROS production and scavenging expressed as Log_2_ fold change (MGS-1/Control). Superoxide Dismutase 1 (*SOD1*), Ascorbate Peroxidase 1 (*APX1*), Glutathione Reductase 1 (*GSR1*), Respiratory Burst Oxidase Homolog (*RBOHD*), Catalase 1 (*CAT1*). Data of panels (E-J) are representative of at least three biological replicates. Density plots are calculated on an average of 20 to 35 measurements. Error bars represent standard deviation. Asterisk indicates significant (*P* < 0.05) differences between MGS-1 exposed plants and controls.

A comparative analysis of maize grown in 30% MGS-1 versus the agarized control substrate (without MGS-1) is shown in Fig. 3. While germination percentage was not significantly different (*P* = 0.538); a significant difference was observed in the plant dry weight (controls, 11.21% ± 3.23; MGS-1, 16.51% ± 2.07; *P* = 0.013). We then assessed several key growth parameters by analyzing density plots (Fig. 3B, C, and D). Compared to controls, MGS-1-grown plants showed a significant reduction (*P* < 0.05) in root length, shoot length, and leaf area. Further analysis of MGS-1-treated plants revealed a consistent, significant (*P* < 0.05) reduction in a range of biochemical metrics compared to the control group. This reduction was observed for total protein content (Fig. 3E), chlorophyll and carotenoid content (Fig. 3F), proline content (Fig. 3G) and total phenolic content (Fig. 3H). A strong reduction of the steady-state H_2_O_2_ content was found in MGS-1 trials (Fig. 3I). We analyzed the expression of genes encoding enzymes involved in ROS production and scavenging. Results are plotted as the Log_2_ fold change in the MGS-1/Control ratio, where positive values indicate upregulation and negative values indicate downregulation (Fig. 3J). In general, MGS-1 up regulated all root genes studied, whereas a strong downregulation was found for Superoxide Dismutase 1 (*SOD1*) and, to a lesser extent, for Ascorbate Peroxidase 1 (*APX1*) and Glutathione Reductase 1 (*GSR1*) in the shoots (Fig. 3J). These genes were chosen because *SOD1*, *APX1*, *GSR1* and *CAT1* encode the primary enzymatic ROS-scavenging network, while *RBOHD* generates apoplastic ROS for signalling^42^. Their differential expression therefore directly informs the redox and developmental trade-offs observed under combined stresses.

### Reduced geomagnetic field (hMF) and MGS-1 impact on maize growth, development, and biochemistry

**Fig. 4:**
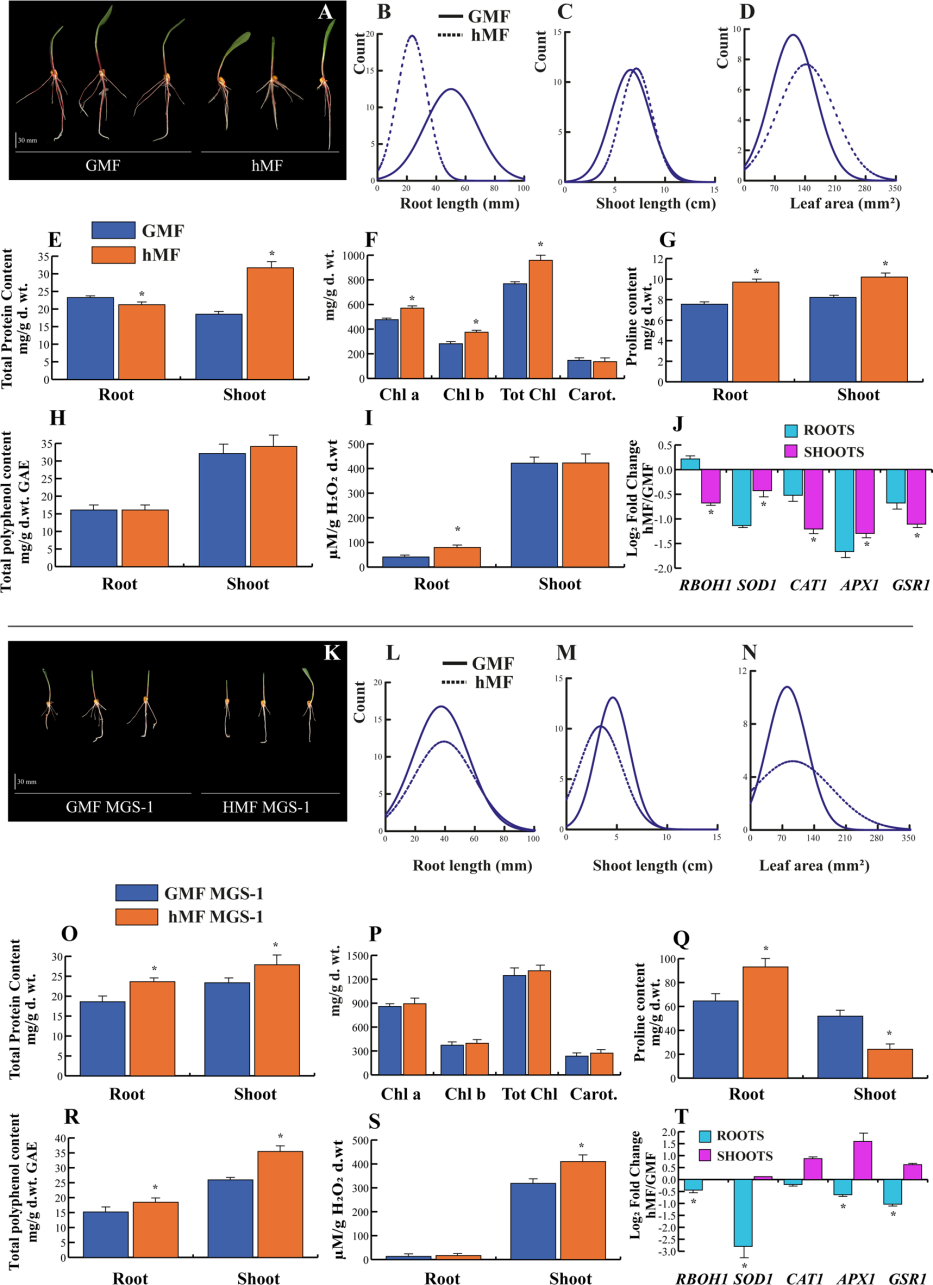
Comparative analysis of *Zea mays* grown in GMF and hMF conditions without and in the presence of MGS-1. **Upper panel**: Morphological and biochemical changes in *Zea mais* grown on half strength MS and Agar in plants grown under hMF conditions as compared to plants grown in GMF (CTRL). **Lower panel**: Morphological and biochemical changes in Zea mais grown on 30% MGS-1 and 70% half strength MS in plants grown under hMF conditions (hMF MSG-1) as compared to plants grown in GMF (GMF MGS-1). (**A**, **K**) Representative plants of the same age grown in treatment and control conditions. (**B**, **L**) Density plot of root length. (**C**, **M**) density plot of shoot length. (**D**, **N**) Density plot of leaf area. (**E**, **O**) Total protein content. (**F**, (**P**) Chlorophyll and carotenoid content. (**G**, **Q**) Proline content. (**H**, **R**) Total polyphenol content. (**I**, **S**) steady-state production of H_2_O_2_. (**J**, **T**) Expression of genes involved in ROS production and scavenging expressed as Log_2_ fold change. Error bars represent standard deviation. Asterisk indicates significant (*P* < 0.05) differences between treatments and controls. Data of panels (E-J and O-T) are representative of at least three biological replicates. Density plots are calculated on an average of 20 to 30 measurements.

Following the assessment of the general effect of MGS-1, we tested the impact of a reduced magnetic field (hMF), simulating Mars’ magnetic conditions, on plant development, biochemical parameters, and gene expression. We observed a significant reduction in root length for plants grown under hMF compared to control (Geomagnetic Field, GMF) conditions (Fig. 4B). However, no significant differences were found between treatments for shoot length (Fig. 4C) or leaf area (Fig. 4D) (see also Fig. S7). Biochemical analysis revealed several notable changes under hMF. In particular, hMF decreased protein content in roots but increased it in shoots (Fig. 4E). In these organs, hMF led to an increase in chlorophyll *a* (Chl *a*), chlorophyll *b* (Chl *b*) and the total chlorophyll content, though carotenoid content remained unchanged (Fig. 4F). The proline content increased in both roots and shoots under hMF conditions (Fig. 4G), whereas total phenolic compounds showed no difference between treatments in either organ (Fig. 4H). The steady-state H_2_O_2_ content increased in roots under hMF, while shoots showed no difference compared to controls (Fig. 4I). In general, hMF downregulated most of the ROS genes studied. The only exception was the slight upregulation of Respiratory Burst Oxidase Homolog D (*RBOHD*) in roots. Notably, a significant modulation was found for *APX1*, which was the most downregulated gene in both organs (Fig. 4J).

We then repeated the experiment by growing maize in the 30% MGS-1 under both hMF and GMF conditions. In the combined MGS-1 + hMF treatment, no significant differences were observed between hMF and GMF conditions for root length (Fig. 4L), shoot length (Fig. 4M), or leaf area (Fig. 4N) (Fig. S8). Regarding biochemical parameters, the combined presence of MGS-1 + hMF increased protein content in both shoots and roots (Fig. 4O) but no significant differences were found in chlorophyll and carotenoid content among the treatments (Fig. 4P). The proline content dramatically increased in roots but significantly decreased in shoots of MGS-1 + hMF maize (Fig. 4Q). Total phenolic content increased in both organs (Fig. 4R), while the steady-state H_2_O_2_ content increased primarily in maize shoots (Fig. 4S). Finally, the combined action of hMF and MGS-1 resulted in a strong downregulation of *SOD1* in roots and an upregulation of *APX1* in shoots (Fig. 4T).

### The simulated reduced gravity of Mars (sMG, simulated fractional gravity (0.38 g)) and the presence of MGS-1 affect maize metabolism and ROS production

**Fig. 5:**
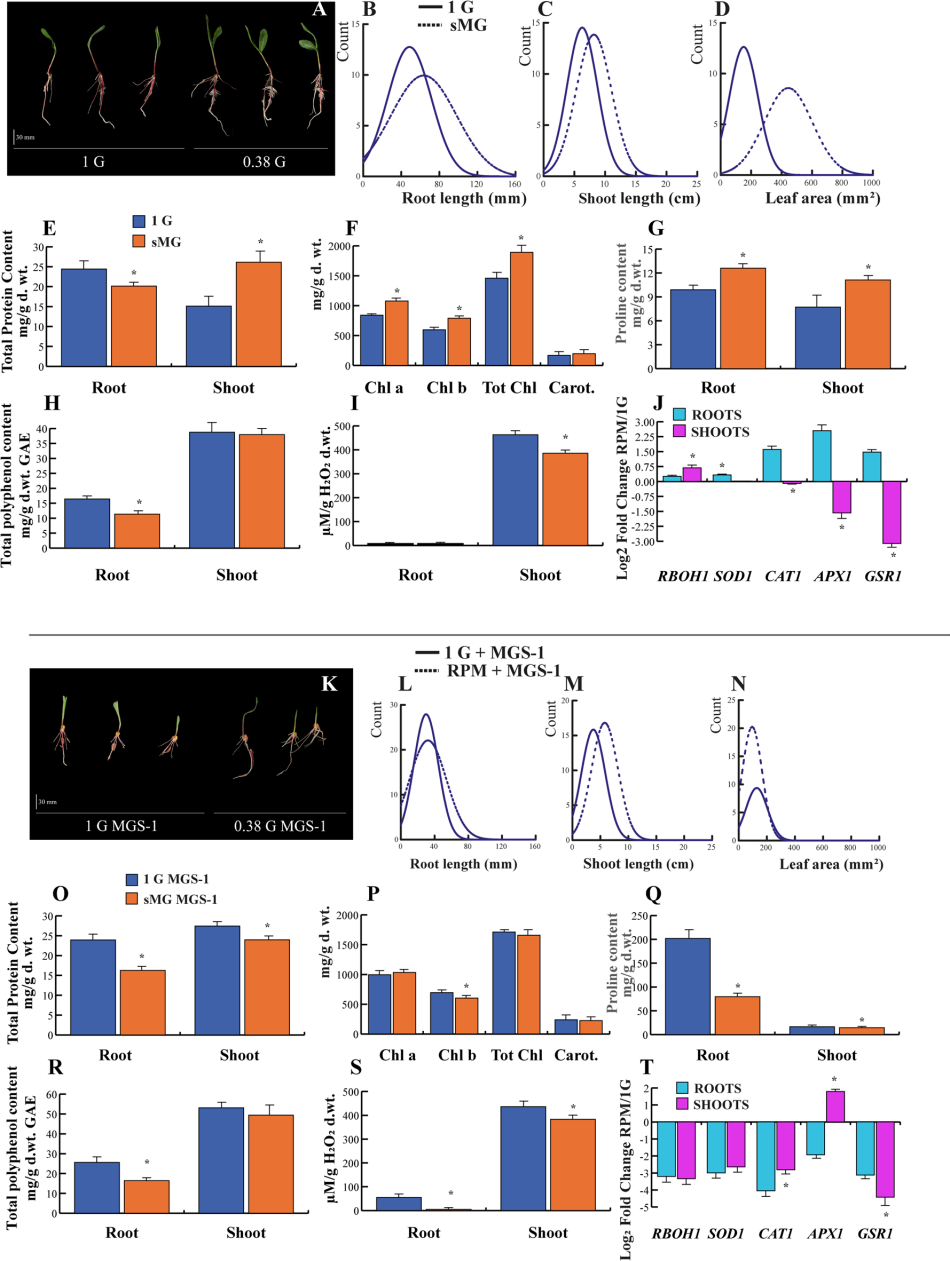
Comparative analysis of *Zea mays* grown in 1G and simulated Mars gravity (sMG, simulated fractional gravity (0.38 g)) conditions without and in the presence of MGS-1. **Upper panel**: Morphological and biochemical changes in *Zea mays* grown on half strength MS and Agar in plants grown under sMG conditions as compared to plants grown in normal gravity (1 G). **Lower panel**: Morphological and biochemical changes in *Zea mais* grown on 30% MGS-1 and 70% half strength MS in plants grown under sMG conditions (sMG MSG-1) as compared to plants grown in normal gravity (1 *g* MGS-1). (**A**, **K**) Representative plants of the same age grown in treatment and control conditions. (**B**, **L**) Density plot of root length. (**C**, **M**) density plot of shoot length. (**D**, **N**) Density plot of leaf area. (**E**, **O**) Total protein content. (**F**, **P**) Chlorophyll and carotenoid content. (**G**, **Q**) Proline content. **H**, **R**) Total polyphenol content. (**I**, **S**) steady-state production of H_2_O_2_. Data are representative of at least three biological replicates. (**J**,** T**) Expression of genes involved in ROS production and scavenging expressed as Log2 fold change. Error bars represent standard deviation. Asterisk indicates significant (*P* < 0.05) differences between treatments and controls.

To assess the effect of reduced gravity, control (1 *g* in GMF conditions) and Mars gravity simulated (sMG, RPM set at 0.38 *g* in GMF conditions), plants were grown in a Random Positioning Machine (RPM) within a light box equipped with LEDs on all six sides (see Movie S1). Growth analysis revealed no significant differences between control and sMG plants in root length (Fig. 5B) or shoot length (Fig. 5C). However, a higher leaf area was measured in the sMG plants (Fig. 5D) (see also Fig. S9).

Metabolic parameters showed modulation under sMG with a decreased root protein content but increased shoot protein content (Fig. 5E). Chlorophyll production increased, while carotenoid synthesis remained unaffected (Fig. 5F). The proline content increased in both organs (Fig. 5G), whereas the total phenolic content decreased in roots and remained stable in shoots (Fig. 5H). We observed a significant decrease in steady-state H_2_O_2_ content in the sMG maize shoots (Fig. 5I). In terms of gene expression, an opposite trend was found for antioxidant genes *CAT1*, *APX1* and *GSR1*, which showed upregulation in roots and downregulation in shoots (Fig. 5J).

The simulant MGS-1 was then tested on plants grown in normal gravity and in sMG (Fig. S10). When comparing control versus sMG plants with MGS-1 present, no significant differences were found in root length (Fig. 5L), shoot length (Fig. 5M), or leaf area (Fig. 5N). The addition of MGS-1 under sMG conditions impacted protein and pigment levels. It significantly reduced leaf (shoot) protein content and exacerbated the reduction of root protein (Fig. 5O). MGS-1 reduced Chl *b* content while leaving the other pigment parameters unaltered (Fig. 5P). Further analysis showed a drastic and significant reduction of proline in both organs upon MGS-1 addition (Fig. 5Q). Conversely, the content of phenolic compounds was reduced only in roots by the MGS-1 treatment (Fig. 5R). ROS production, specifically steady-state H_2_O_2_ content, was reduced by sMG in the presence of MGS-1, with a stronger effect observed in roots (Fig. 5S). Finally, MGS-1 downregulated almost all genes in both organs, with the only exception being *APX1*, which was upregulated in shoots (Fig. 5T).

### The simulated Mars reduced gravity, hypomagnetic fields and the presence of MGS-1 impact on maize PIN gene expression

Having assessed the effects of both hMF and sMG on root morphology, we evaluated the gene expression of three members of PIN gene family involved in auxin efflux: *ZmPIN1a*, localized predominantly basally (toward root tip) in epidermis cells; *ZmPIN1b*, an auxin-efflux facilitator involved in root gravitropism, and *ZmPIN1c*, responsible for the lateral relocation of auxin efflux^43^.

**Fig. 6:**
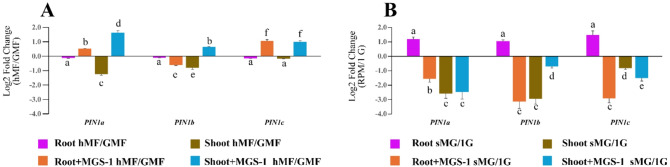
Effects of MGS-1 in the presence of reduced magnetic field (hMF, panel **A**) and simulated Mars gravity (sMG, panel **B**) on the expression of *PIN* genes in roots and shoot of *Zea mays*. Error bars represent standard deviation. Different letters indicate significant (*P* < 0.05) differences.

In the hypomagnetic field condition (hMF/GMF), the expressions of *ZmPIN1a*, *ZmPIN1b*, and *ZmPIN1c* were unaffected in roots, but *ZmPIN1a* and *ZmPIN1b* were downregulated in shoots. The presence of the Mars simulant regolith MGS-1 completely reversed this pattern in both tissues. In roots, MGS-1 exposure caused a significant upregulation of *ZmPIN1a* and *ZmPIN1c*, while *ZmPIN1b* showed a slight downregulation. Similarly, in the shoots, MGS-1 reversed the initial downregulation, leading to a upregulation of all *PIN* genes (Fig. 6A).

Reducing gravity to Mars conditions (sMG) prompted a significant upregulation of all *PIN* genes in roots and a marked downregulation in shoots. The addition of MGS-1 completely reversed the sMG effect in roots, leading to a strong downregulation of all *PIN* genes, with the most severe effect observed for *ZmPIN1b*. In the shoots, the addition of MGS-1 had no significant effect on the expression of *ZmPIN1a* compared to the control, but it did result in a notable lessening of downregulation for *ZmPIN1b* expression and a more pronounced downregulation of *ZmPIN1c* (Fig. 6B).

## Discussion

The successful establishment of agriculture on Mars centres on understanding how terrestrial organisms, such as maize (*Zea mays* L.), integrate and respond to the planet’s unique combination of environmental stressors: reduced gravity, a near-absent magnetic field (hMF), and chemically complex regolith^14^, among others. This study employed a multidisciplinary approach by combining mineralogical, morphological, biochemical, and gene expression analyses to characterize maize adaptive responses. It is conceivable that any Mars farming will use greenhouses employing Earth-like parameters, such as Earth atmospheric pressure and composition. Our findings collectively demonstrate that, under an Earth atmosphere, while both hMF and sMG induce specific, organ-polarized changes in maize metabolism, the chemical complexity of the MGS-1 acts as the dominant environmental factor, imposing a pervasive stress that frequently overrides or severely modulates the plant’s response to the Mars environmental stresses.

### The Martian regolith is a limiting factor for maize growth

The Martian regolith poses a critical limitation for maize growth, as evidenced by the MGS-1, a high-fidelity analog of global basaltic Martian regolith^11^ designed for in situ resource utilization (ISRU)^44^. Mineralogical analyses revealed a complex matrix dominated by phases that potentially interact with abiotic factors (e.g., T, pH, pCO₂, pO₂) and biotic systems (maize rhizosphere), yet their dissolution kinetics and bioavailability often hinder plant viability.

Surface alteration of anorthite through agitation, wet processing, microwave heating, and crushing^11^ accelerates its dissolution at pH 6.0, potentially exacerbating nutrient imbalances through abrasion-enhanced reactivity^45^. Anorthite dissolution releases Ca/Al, potentially causing plant toxicity (e.g., root inhibition via Al_3_^[+ 46^). Pyroxenes dissolve through surface-controlled etching, forming lens-shaped pits characteristic of chain silicates under terrestrial conditions^47^, but this may release toxic ions on Mars, restricting root development. Pyroxenes etch pits also increase ion leaching, disrupting Mg/Fe uptake and potentially affecting root development via auxin imbalance^48^. The olivine persistence implies limited water exposure^49^, although factors such as carbonates, aqueous SiO₂, grain coatings, ionic strength, fayalite-forsterite ratios, grain size, and hydrodynamics^50,51^ could unpredictably alter Fe and Mg availability, risking deficiency or toxicity. Olivine persistence also limits Mg release, potentially causing photosynthesis impairment^52^.

Gypsum provides S but underscores broader solubility challenges. The epsomite limited content suggests a loss of this phase during preparation due to high solubility and possible phase transitions^5^. SO₄ mobility, influenced by clay content, mineral type, pH, cations, and Al/Fe oxides^53^, may inhibit Fe-OC interactions via ligand exchange disruption, particularly under reducing conditions where Fe²⁺ dissolves^54,55^, thereby compromising soil OC preservation and nutrient cycling. Because roots are the the primary interface between plants and soil, compromised soil OC input may affect root turnover and exudation^56^.

Fe-oxides (e.g., magnetite and hematite) are widespread, but the ferrihydrite instability^57^ raises the possibility of Fe remobilization. The Fe solubility varies with pH, light, O₂, and acids^58^, while weathering converts labile forms to crystalline ones^59^, reducing bioavailability. Photoreduction and organic ligands^60^ may promote dissolution, but nanoscale Fe-OC affinities^61^ form stable aggregates^62,63^, contributing to the OC sequestration^64,65^. Amorphous Fe-Mg-Si particles suggest organo-metallic re-precipitation or resolution limits, further complicating Fe dynamics. These effects might interfere with regulatory hubs that link Fe status to cell-wall remodeling and crop resilience^66^.

Diatomite improves water and nutrient retention as well as structure^67^, yet it could contribute to Si release or metal immobilization depending on impurities and pH. Quartz dissolution depends on pH, cations, organic acids, and Al/Fe (not structural defects), potentially mobilizing Si but altering regolith stability. It has been shown that natural variation in plant-available Si influences plant performance^68^. Mica reduces cohesion and increases brittleness, despite improving friction^69^, thus weakening regolith structure for root penetration^70^.

MGS-1 alone fails to support plant growth and, even with added nutrients, requires modifications^13,71^. It induces severe ionic, osmotic, and nutrient stresses, suppressing defence compounds (proline, phenolics, H₂O₂) and indicating metabolic stasis rather than an adaptive response, mirroring issues in other simulants^72^. Altered pH/minerals inhibit nutrient cycling, thus suppressing growth^13^.These findings challenge the viability of ISRU, necessitating targeted amendments to mitigate mineral toxicities and enhance bioavailability for sustainable Martian agriculture.

### Interaction of hypomagnetic field and regolith: PIN reversal

The Martian environment presents unique biophysical and chemical challenges to plant life, notably a hypomagnetic field (hMF)^73^ and mineral-rich regolith (MGS-1)^8^. Our results demonstrate that the presence of regolith dramatically alters the transcriptional and physiological disturbances induced by hMF, often reversing rather than simply mitigating them.

Under hMF alone, reduction of root length confirms that the lack of a MF acts as a specific biophysical stressor on subterranean growth. While varying magnetic flux densities have been shown to influence maize root development by altering cell expansion in both acropetal and basipetal directions^74^ or increasing growth rates^75^, the near-null field of hMF appears inhibitory. This morphological defect is linked to disrupted auxin transport (downregulation of *ZmPIN1a* and *ZmPIN1b* in shoots) and altered ROS signalling, both of which impair auxin-mediated gravitropism and root elongation. Auxin efflux carrier genes, which are expressed ubiquitously but regulated differentially across organs^76^, play a central role in maintaining auxin homeostasis.

In contrast, the addition of MGS-1 abolished hMF-induced root shortening, a recovery paralleled by a dramatic reversal in *PIN* expression. The combined MGS-1 + hMF altered the gravitropism and lateral root initiation^77^, suggesting that the chemical and osmotic stress of MGS-1 acts as a dominant signal, triggering robust acclimatory mechanisms that override hMF-induced signalling disruptions.

The plant’s anabolic and defensive capacities also improved under dual stress. While hMF alone caused an inverse protein correlation between organs, the dual treatment increased protein content globally. Among plant’s adaptations to stress, modulation of regulatory proteins play an essential role^78^, and abiotic stress is known to increase the content of specific proteins^79^ Crucially, the antioxidant response was contingent upon the regolith: hMF alone suppressed the major antioxidant *APX1*, consistent with known ROS production patterns in hypomagnetic conditions^80^. Conversely, the presence of MGS-1 led to a complete regulatory reversal, characterized by the clear upregulation of *APX1* in the shoots. Using regolith as a plant substrate causes oxidative stress^16,81^ and plants have evolved antioxidant defence mechanisms to fight excessive accumulation of ROS, including SOD, CAT, and APX^82^. The latter scavenges H_2_O_2_ from plant chloroplasts and the cytoplasm and improves resistance to oxidative stress while enhances stress resistance^83^. Proline accumulation supports osmoprotection and cellular redox balance, H₂O₂ modulation reflects photo-oxidative defence, protein content indicates anabolic capacity, and chlorophyll/carotenoid levels together with polyphenols underpin photoprotection and antioxidant buffering. These findings suggest that regolith-induced stress is a necessary catalyst for activating the plant’s established defensive machinery in the Martian environment.

### Adaptive trade-off: simulated fractional gravity (0.38 g) and regolith stress

The combination of simulated Martian gravity and soil chemistry forces a distinct developmental and metabolic trade-off in maize, suggesting a reduced energetic cost for maintaining shoot architecture in lower gravity environments.

At the molecular level, sMG triggered a strong auxin response in roots to maintain gravitropic signalling and a reduced ROS levels in shoots. Given that ROS play an active role in the gravitropic responses^84^, these reduced shoot ROS levels might be directly correlated to the observed increased leaf area. However, the sMG + MGS-1 combination proved severely detrimental. The chemical toxicity of MGS-1 not only abolished the gain in leaf area but led to systemic metabolic depletion. The drastic reduction in proline and an exacerbated loss of total protein content in both organs indicates that the combined sMG + MGS-1 stress compromises both anabolic processes and the maintenance of crucial defence reserves. This adaptive failure was confirmed by the complete reversal of auxin transport signalling. While experiments on the International Space Station (ISS)^85^ and in simulated microgravity^86^ have shown that auxin polar transport is typically inhibited under microgravity, the combined sMG + MGS-1 stress caused a profound downregulation of all genes involved in auxin carriers in the roots. This suppression suggests a clear developmental trade-off: the plant halts the energy-intensive processes of gravitropic signalling and lateral root development to prioritize immediate survival under severe constraints^87^. Ultimately, the sMG + MGS-1 environment forces the plant into a survival mode characterized by metabolic exhaustion and the suppression of complex developmental programs.

### The dominant role of regolith in determining metabolic trade-offs

While MGS-1 acts as the primary driver of the plant’s response, the specific biophysical challenge (hMF versus sMG) dictates the final metabolic trade-off. In both combined treatments (hMF + MGS-1 and sMG + MGS-1), the presence of regolith forces the plant to prioritize baseline morphology, effectively nullifying the growth modulations observed under individual biophysical stressors.

MGS-1 is known to increase proline and ROS levels in crops^12^. Interestingly, both combined environments exhibited a conserved commitment to protecting the photosynthetic apparatus pointing to a universal survival priority in Martian-simulated conditions. However, core energy management diverged sharply between the two treatments. In hMF + MGS-1, the plant maintained a functional anabolic state and retained signalling plasticity, while increasing H_2_O_2_ steady state. Proline functions here as a compatible osmolyte that stabilises proteins and membranes under osmotic stress and also acts as a ROS scavenger^88^, supporting cellular redox balance and nitrogen storage for recovery. In contrast, in sMG + MGS-1 the plant suffered systemic metabolic depletion, indicating a compromised capacity for anabolic synthesis. Reduced proline levels reflect failure of osmoprotection and nitrogen remobilisation, while lowered chlorophyll and carotenoid contents signal impaired photosynthetic efficiency and photoprotection. Polyphenols, which normally serve as non-enzymatic antioxidants and signalling molecules^89^, are also suppressed, weakening the plant’s capacity to quench excess ROS and modulate stress signalling. H₂O₂ itself plays a dual role: at moderate levels it acts as a signalling molecule that coordinates defence and growth, yet at high concentrations it becomes cytotoxic^90^; its differential accumulation therefore explains the organ-specific trade-offs observed. The ROS-signalling gene *RBOHD* is central to this response, producing apoplastic superoxide that is rapidly converted to H₂O₂ and participates in ethylene-ROS crosstalk, cell-wall loosening, and root-growth regulation^91^. Its strong downregulation under sMG + MGS-1 therefore contributes to the observed transcriptional shutdown of *PIN* genes and the switch from adaptive growth to survival-oriented stasis.

### Limitations of the experimental system

The use of 30% MGS-1 in agar with MS media positions the regolith more as an additive than a primary substrate. This choice was intentional to enable initial viability assessments in a controlled, semi-solid system suitable for RPM integration and short-term stress simulations, as higher concentrations (e.g., > 50%) are often prohibitively toxic due to perchlorates (0.5–2 wt%), salinity, and nutrient deficits, leading to germination failure or severe growth inhibition^92,93^. A recognized constraint of the present study is the use of standard 120-mm vertical square Petri dishes for the gravitropism assays. In 7-day-old maize seedlings, shoots inevitably become crowded against the lid, which can locally elevate ethylene concentrations through mechanical contact and restricted headspace gas exchange. Clinorotation and RPM rotation themselves have been shown to stimulate ethylene biosynthesis in several species via mechano-stimulation and altered fluid dynamics^94^. To minimise this artefact we used gas-permeable plate seals, provided uniform LED illumination from all six sides of the RPM enclosure, and continuously monitored expression of *RBOHD*, a key gene at the ROS–ethylene signalling interface^95^. The fact that RBOHD expression patterns remained treatment-specific (strongly downregulated in sMG + MGS-1 roots, upregulated in hMF + MGS-1 shoots) and that regolith-free 1 *g* and hMF controls grown in identical plates did not exhibit the same systemic metabolic collapse strongly indicates that the dominant driver of the observed phenotypes is the chemical toxicity of MGS-1 rather than plate-induced ethylene stress.

### Synthesis of results: a unified mechanistic model

**Fig. 7:**
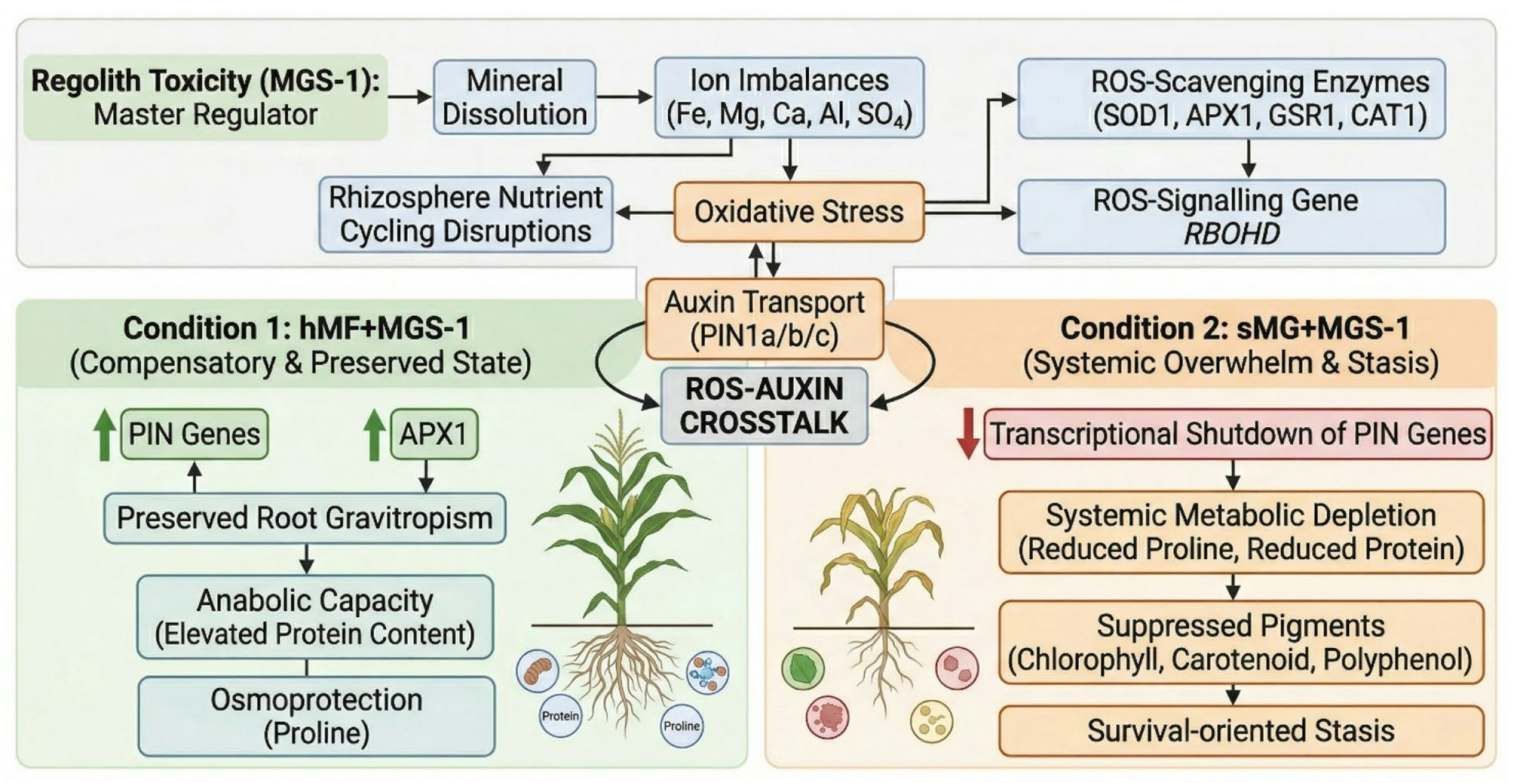
A proposed mechanistic model illustrating MGS-1 regolith toxicity in *Zea mays* under hypomagnetic fields (hMF) and simulated Martian gravity (sMG). MGS-1 dissolution drives ion imbalances (Fe, Mg, Ca, Al, SO₄), leading to oxidative stress and disruptions in nutrient cycling. This oxidative stress modulates ROS-scavenging enzymes (e.g., APX1) and the signalling gene *RBOHD*, which in turn interacts with auxin transport (*PIN* genes) through “ROS-auxin crosstalk.” Condition 1 (hMF + MGS-1, light green panel) details a compensatory, preserved state where upregulated *PIN* genes and *APX1* preserve root gravitropism and anabolic capacity. In contrast, Condition 2 (sMG + MGS-1, light orange panel) exhibits systemic metabolic depletion and survival-oriented stasis due to overwhelmed defences. Ultimately, regolith chemistry overrides biophysical cues by altering the coordination between growth, defence, and resource allocation via ROS-auxin interactions.

Collectively, the data reveal a coherent mechanistic framework in which MGS-1 regolith toxicity acts as the master regulator. Mineral dissolution and ion imbalances (Fe, Mg, Ca, Al, SO₄) disrupt rhizosphere nutrient cycling and generate oxidative stress, which in turn modulates ROS-scavenging enzymes (SOD1, APX1, GSR1, CAT1) and the ROS-signalling gene *RBOHD*. This redox imbalance interacts with auxin transport (PIN1a/b/c) to dictate organ-specific trade-offs: under hMF + MGS-1, compensatory upregulation of *PIN* genes and *APX1* preserves root gravitropism and anabolic capacity (elevated protein content), while proline supports osmoprotection. In contrast, sMG + MGS-1 overwhelms these defences, causing systemic metabolic depletion (reduced proline and protein), transcriptional shutdown of *PIN* genes, and suppressed chlorophyll/carotenoid/polyphenol levels, forcing the plant into a survival-oriented stasis. Thus, regolith chemistry overrides biophysical cues by modulating the ROS-auxin crosstalk that normally coordinates growth, defence, and resource allocation. Figure 7 summarizes the mechanistic model.

In conclusion, this study underscores the Martian regolith as a profound barrier to extraterrestrial agriculture, revealing how its chemical and structural complexities fundamentally undermine crop viability. By dominating over biophysical stressors like hMF and sMG, the regolith exposes critical vulnerabilities in plant resilience, challenging the feasibility of unamended ISRU for sustaining human missions.

These findings also point to a divergence in plant-environment interactions: regolith exposure can buffer hMF effects, fostering metabolic adaptability and auxin-mediated developmental plasticity, yet it exacerbates sMG constraints, driving energy reallocation toward bare survival at the expense of growth and signalling pathways. This not only highlights the regolith’s role in dictating organ-specific trade-offs but also emphasizes the broader implications for space biology, demonstrating how combined extraterrestrial factors could reshape evolutionary adaptations in terrestrial species, with parallels to extreme Earth environments like arid or contaminated soils. While the use of RPMs for simulating partial gravity, such as Martian levels, remains somewhat controversial due to potential artifacts like pole bias in standard two-frame designs, our optimized parameters (e.g., rotation rates > 30 deg/s, sample positioning < 10 cm from centre) align with recent kinematic validations and guidelines that confirm RPM fidelity for reduced-gravity analogs, including Mars^96^.

To advance sustainable farming beyond Earth, our work stimulates the search for innovative ISRU interventions. Standardizing simulants and integrating closed-loop systems will be crucial to translating these insights into resilient crop systems, paving the way for self-sufficient habitats on Mars and other celestial bodies^92^.

## Methods

### Regolith simulants and plant material

MGS-1 was acquired from Space Resources Technologies (Oviedo, FL, USA). Supplementary Table S1 provides the compositional information for MGS-1 as supplied by the vendor. Maize (*Zea mays* L. cv. DKC6980) caryopses were sourced from Dekalb, Mestre, Italy.

### Powder X-ray diffraction (PXRD) analysis

PXRD was utilized to characterize the mineralogical phases of the MGS-1. One-gram of the MGS-1 was mechanically ground for 5 min. at 40 oscillations per second (Pulverisette 23, Fritsch Idar-Oberstein, Germany) before being positioned in an Al-based sample holder using the “side-loading” technique. Two distinct PXRD measurements were performed: a preliminary qualitative analysis was conducted to identify the crystalline phases, while a quantitative measurement was performed to confirm the identity and quantify the crystalline phases identified in the preliminary qualitative analysis, as well as quantifying both the amorphous material percentage with respect to the total analyzed mass. For the quantitative analysis, the MGS-1 was spiked with 20 wt% of high purity α-Al_2_O_3_ to calculate the amorphous fraction using the Rietveld-Reference Intensity Ratio (RIR) method^97^. Qualitative PXRD data were collected on a Miniflex 600 diffractometer (Rigaku, Tokyo, Japan) equipped with a Cu–Kα1 radiation source (λ = 1.54055 Å, 40 mA, 15 kV), fixed divergence slits, and a multistrip D/TEX Ultra detector with a resolution of < 200 eV. Data acquisition spanned the 3–70° 2*θ* range, with a step size of 0.02°, and a counting time of 0.7 s per step. A correction for the non-negligible Fe fluorescence generated by the Cu radiation was applied. The resulting spectra were processed using PDXL software (Rigaku, Tokyo, Japan). Quantitative PXRD data were collected using a SmartLab diffractometer (Rigaku) equipped with a Cu–Kα1 radiation source (λ = 1.54055 Å, 40 mA, 30 kV), fixed divergence slits, and an area HyPIX detector. Data were collected over the 3–70° 2*θ* range, with a step size of 0.02°, and a counting time of 0.4 s per step. An XRF reduction correction was applied along with the qualitative measurement. The obtained spectra were subsequently analyzed with GSAS-II software^98^.

### Scanning electron microscopy-energy dispersive X-ray spectroscopy (SEM-EDXS) analysis

SEM-EDXS was used for micro-scale morphological and chemical investigation. The analyses were performed using a SEM Tescan Vega 3 (Tescan, Brno, Czech Republic) operating at high vacuum (~ 6.740 × 10^−2^ Pa), at a voltage of 30 keV and at a beam current of 1 nA, equipped with an EDXS detector (Oxford Instruments, Oxfordshire, England) managed by the AztecONE software (version 6.0). Samples were prepared by gently air-blowing the powder directly onto SEM Al stubs covered with C tape (Media System Technologies, Macherio, Italy). The samples were then coated with C using a JEOL Jec-530 Auto Carbon Coater (JEOL, Tokyo, Japan). Pictures were acquired at various magnification, and EDXS spectra were collected on selected spots in the observed regions of interest (ROIs). Each spectrum was collected with a variable live time ranging from 20 s to 50 s.

### Scanning/transmission electron microscopy-EDXS (S/TEM-EDXS) analysis

S/TEM-EDXS was employed for morphometric, mineralogical and physicochemical characterization. The analysis was conducted using a JEOL ARM 200 CF operating at 80 keV and equipped with an EDXS detector (Centurio 100 mm^2^, JEOL). Samples were first mechanically ground for 5 min at 40 oscillations per s (Pulverisette 23, Fritsch Idar-Oberstein, Germany), then suspended in acetone, and placed in an ultrasonic bath for 10 min. A droplet of this suspension was then transferred via pipette onto a TEM 300 mesh lacey grid (TED PELLA, INC. Canada, USA). For each observed particle and aggregate, low–resolution (LR) and high–resolution (HR) images were acquired. Images were processed with the Gatan Digital Micrograph software (version 3.62.4983.0, California, USA), which was used to obtain Fast Fourier Transform (FFT) diffraction patterns and filtered images by applying the Inverse Fast Fourier Transform (IFFT) function. An accurate mineral phase identification was performed by combining the PXRD spectra, the chemical composition data (from SEM-EDXS and S/TEM-EDXS spectra), and the HR images (and/or related IFFT images) by S/TEM. Cross-reference was performed using established data repositories such as: the Mindat, Webmineral, Mineralienatlas, and RRUFF database^99–101^.

### Plant growing conditions and growth parameters

Maize caryopses were initially rinsed with distilled water to remove the commercial antifungal coating, followed by surface sterilization with 0.7% NaClO for 10 min, and a final rinse with sterile water. The control substrate was a half-strength Murashige and Skoog (MS) basal medium^102^, buffered at pH 6.0 with MES (2-morpholinoethanesulfonic acid) and solidified with 0.8% Plant Agar (Duchefa Biochemie, Milan). MS was chosen for its suitability in agar-based tissue culture, whereas the Hoagland solution is normally used for hydroponics/soils. After different trials with increasing MGS-1 concentrations, the MGS-1 simulated regolith substrate was prepared by dispersing MGS-1 into the control MS solution at a proportion of 30% (v/v of MGS-1), being higher concentrations toxic for plant growth. This concentration was also chosen to mimic additives in early ISRU^13^. This translates to a 70:30 ratio of agarized MS solution to MGS-1, based on the volume of MS solution and the mass of MGS-1. The pH of the final mixture was adjusted to 6.0 using concentrated H_2_SO_4_. Both the control and MGS-1-containing solutions were autoclaved. While the medium was still warm, it was vigorously shaken to ensure the homogeneous dispersion of MGS-1 before being poured into squared Petri dishes under sterile conditions. Eight sterilized caryopses were seeded in four 12 × 12 cm Petri dishes sealed with gas permeable 3 M tape (Micropore™), and the experiments were replicated three times. Each treatment consisted of *n* = 3 biological replicates, each consisting of 4 plates with 8 plants per plate (total 96 plants per group across factors). To assess the effects of MGS-1 on maize, seedlings were grown under controlled conditions: 22 ± 1.5 °C, 60% relative humidity, and a 16/8 h light/dark photoperiod. For experiments involving the assessment of hypomagnetic fields and simulated Mars gravity, maize seedlings were grown vertically under the same temperature, humidity, and photoperiod conditions. White LED lighting provided a uniform photosynthetic photon flux density (PPFD) of 120 µmol m⁻² s⁻¹. For plants growing in the Random Positioning Machine (RPM, see below), a custom-built LED light box was constructed to prevent shading caused by the rotation of the system. LEDs were distributed on the six inner sides of the box to maintain a consistently even light distribution of 120 µmol m⁻² s⁻¹. An internal fan regulated air circulation and maintained a constant temperature within the box. Both RPM and its corresponding 1 *g* control experiments were conducted simultaneously under these identical conditions.

### Hypomagnetic field (hMF) generation system and plant exposure

The experiments were conducted at a location with typical Northern Hemisphere GMF values (45°0’59” N and 7°36’58” E coordinates). Hypomagnetic field (hMF) was generated as previously described^103^. The magnetic field (MF) inside the plant exposure chamber was monitored in real-time using a three-axis magnetic field sensor (model Mag-03, Bartington Instruments, Oxford, U.K.)^31^. The triaxial Helmholtz coils system maintained the MF constant inside the chamber at an hMF intensity of ∼40 nT by precisely controlling the current applied through each coil pair^30^. Plants were positioned at the geometric center of this system for exposure to hMF (~ 40 nT). Plants were harvested after 7 days of exposure, extracted with liquid nitrogen and stored at -80 °C.

### Martian gravity simulation by random positioning machine

Simulated Martian reduced gravity (sMG, simulated fractional gravity (0.38 g)) was achieved using a Random Positioning Machine (RPM; Yury, Meckenbeuren, Germany). The RPM simulates fractional gravity by constantly changing the sample’s orientation relative to the Earth’s gravity vector. When the integration of these vectors over time results in a non-zero net vector (e.g., 0.38 g), it mimics the directional cue of partial gravity. This is achieved by specific software algorithms (e.g., the ‘random walk’ or ‘frame-switching’ modes) rather than physical shielding of gravity^27,104^. The RPM offsets Earth’s 1 *g* via continuous reorientation, preventing sustained gravitational alignment. The RPM minimizes the influence of Earth’s directional gravity and shear forces (especially near its center of rotation) by continuously and randomly reorienting the samples and this generates effects similar to reduced gravity^27,105^. Therefore, The RPM does not offset Earth’s constant 1 *g* gravity with an equal-and-opposite mechanical force. Instead, it achieves a time-averaged reduced gravity vector by rapidly randomising sample orientation relative to the Earth gravity vector^104^. When rotation rates exceed the biological response time of plant statoliths (typically 1–10 s, an estimated threshold for physiological integration, rather than a universal constant for all cell types)^106,107^, the net directional cue experienced by the cells averages to the target partial *g* level (0.38 *g* for Mars) over time^27,94,96,105^. Validation comes from kinematic models and empirical tests showing fidelity to spaceflight effects^96^. We used an outer frame rotation of 35 deg/s and inner frame 15 deg/s slower (20 deg/s), sample size < 10 cm from centre, and duration > 25 min per run, aligning with validated guidelines^96^. No offsetting forces dominate shear; contributions are quantified as < 5% error according to^96^. Maize caryopses were sown in the center of the Petri dishes, aligned with the RPM’s geometric center. The rotation speed in three dimensions was controlled by the Yury software, operating the RPM at 0.38 *g* with continuous rotation to simulate Martian surface gravity. Media density (~ 1.05 g/cm³ for 30% MGS-1 agar mix), viscosity (~ 1.2 Pa·s), cell size (root tips ~ 10–50 μm), and porosity (agar ~ 90%) were considered; shear stress is low (~ 0.1–1 Pa, below plant damage thresholds). Coriolis forces are negligible at these rates (< 0.01 *g* equivalent), and centrifugal forces are minimal ( < < 0.01 *g* at center). These do not significantly confound gravity simulation, as validated in plant and animal studies^108,109^. Control plants were grown under identical conditions but without RPM rotation. Plants were harvested after 7 days of exposure, extracted with liquid nitrogen and stored at -80 °C.

### Plant growth parameters

After 7 days, the following plant growth parameters were assessed: percentage of germination, stem length, root length, root angle, and dry weight. Morphological data were acquired by photographing the plates and subsequently analysed using ImageJ software^110^. Each experimental treatment included a minimum of three biological replicates.

### Total protein, chlorophyll, carotenoids, phenols, H_2_O_2_and proline quantification

One hundred milligrams of fresh leaves and roots from each experiment were ground in liquid nitrogen (N_2_). For protein and proline determination, tissues were extracted with 50 mM sodium phosphate buffer (pH 7.0) along with 100 mg polyvinylpolypyrrolidone (PVPP) to bind phenolic compounds. All subsequent steps for protein and proline extraction (homogenization, centrifugation, and storage) were strictly performed under cold conditions (4 °C). Total protein content was measured in triplicate using a Protein Assay Kit (Thermo Fisher Scientific, Waltham, MA, USA) following the manufacturer’s instructions. Proline extraction was performed using 70% ethanol, with overnight incubation. Proline determination was performed with the ninhydrin reaction according to the method of Friedman^111^. Acid-ninhydrin assay targets proline’s pyrrolidine ring and has been widely validated in plants with minimal overlap. Chlorophyll (Chl) and carotenoid (Car) content were extracted and quantified based on a modified protocol from Lichtenthaler^112^. Specifically, 100 mg of ground leaf material was extracted with 95% ethanol, and Chl *a*, Chl *b*, and Car were measured spectrophotometrically (Shimadzu UV1280, Kyoto, Japan) at 664 nm, 649 nm, and 470 nm, respectively. Lichtenthaler^112^ equations correct for overlap at 663, 645, and 470 nm. The total phenolic content was determined using the Folin–Ciocalteu’s reagent method, which involves the reduction of phosphotungstic-phosphomolybdic acid to blue pigments in an alkaline solution, as previously established^113^. Steady-state hydrogen peroxide (H_2_O_2_) quantification required tissue homogenization and extraction steps performed under cold conditions (4 °C) to minimize degradation or continued production of the molecule. The extraction buffer used Trichloroacetic Acid (TCA) to effectively precipitate proteins and stop enzymatic activity, thereby stabilizing the H_2_O_2_. H_2_O_2_ content was measured in triplicate using the MAK311 Peroxide Assay Kit (Sigma-Aldrich, St. Louis, MO, USA), with quantification based on a standard H_2_O_2_ curve, as previously reported^31^. All experiments were performed in triplicate. Although the spectrophotometric assays used here incorporate established correction equations for spectral overlap, minor contributions from matrix complexity or protein-pigment interactions cannot be entirely excluded and represent a recognised limitation of these widely used approaches.

### mRNA extraction and real-time PCR

Total RNA was isolated and purified from three independent biological replicates of maize tissues (*N* = 6 different plants) using Peqlab PeqGOLD TriFast reagent (VWR Avantor, Radnor, PA, USA). A BioSpec-nano nanospectrophotometer (Shimadzu, Kyoto, Japan) was used to quantify the extracted RNA. Five hundred nanograms (500 ng) of total RNA were utilized to synthesize cDNA using qScript Ultra Supermix (Quantabio, Beverly, MA, USA), following the manufacturer’s instructions. Quantitative Reverse Transcription PCR (qRT-PCR) assays were carried out on a QuantStudio 3 Real-Time PCR System (Applied Biosystems, Foster City, CA) using Perfecta SYBR Green Fastmix (Quantabio, Beverly, MA, USA) with ROX as an internal loading standard. The 10 µL reaction mixture consisted of 5 µL 2X Perfecta SYBR Green Fastmix qPCR Master Mix, 0.25 µL cDNA, and 0.01 nmol of primers (Integrated DNA Technologies, Coralville, IA, US). Non-template controls (water template) were included. PCR conditions for all primers were: Hold stage: 2 min at 50 °C, 10 min at 95 °C; PCR stage: 40 cycles of 15 s at 95 °C, 1 min at 60 °C;10 s at 72 °C; Melt curve stage: 1 min at 60 °C, 1 s at 95 °C. All runs were followed by a melting curve analysis from 55 °C to 95 °C. All amplification plots were analyzed to obtain Cycle threshold (C_t_) values. The *elfα2* (elongation factor α-2) was used as reference gene. The ΔΔ^Ct^ method was used to analyze C_t_ values. Primers used for real-time PCR were designed using the Primer3 (https://primer3.ut.ee/) software and are reported in Supplementary Table S2.

### Statistical analysis

Each statistical analysis was based on a minimum of three biological replicates. Data are presented as mean values, with fold changes relative to the control, and error bars representing the standard error (SE). To evaluate the significance of differences between treatments, a one-way ANOVA was performed, followed by the Tukey test for post-hoc comparisons.

## Supplementary Information

Below is the link to the electronic supplementary material.

Supplementary Material 1

Supplementary Material 2

## Data Availability

All data supporting the findings of this study are available within the paper and its Supplementary Information.
