# Supplementary material for: Maize (Zea mays L.) survival on Mars depends on regolith’s chemical composition rather than reduced gravity or lack of magnetic field
Source: Sci Rep. 2026 Apr 28;16:19504. doi: 10.1038/s41598-026-50840-4 (PMC13287604; doi:10.1038/s41598-026-50840-4)
Supplement: Supplementary file 2 — Supplementary Material 2 [file 41598_2026_50840_MOESM2_ESM.pdf]

Supplementary Figures and Tables

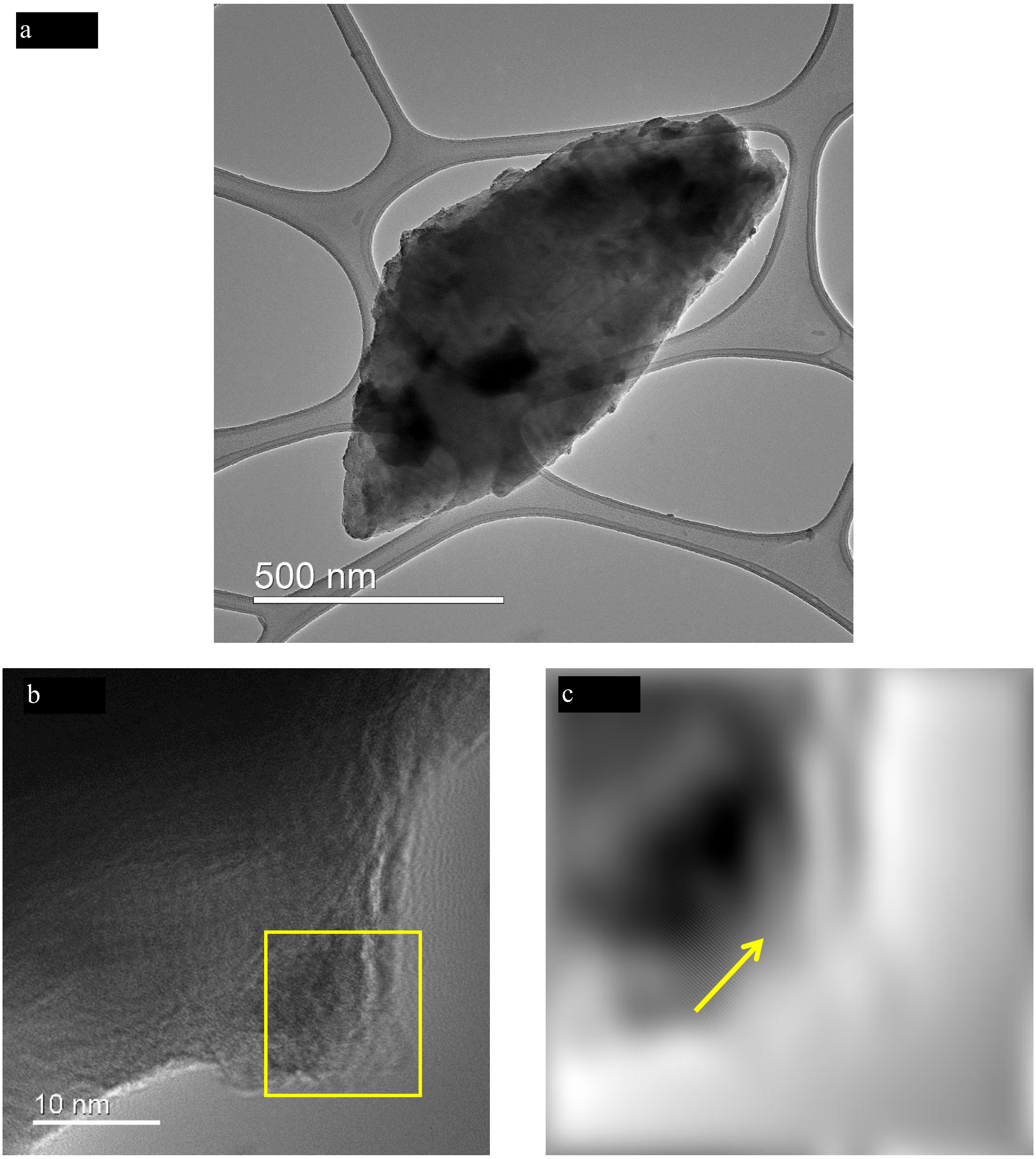

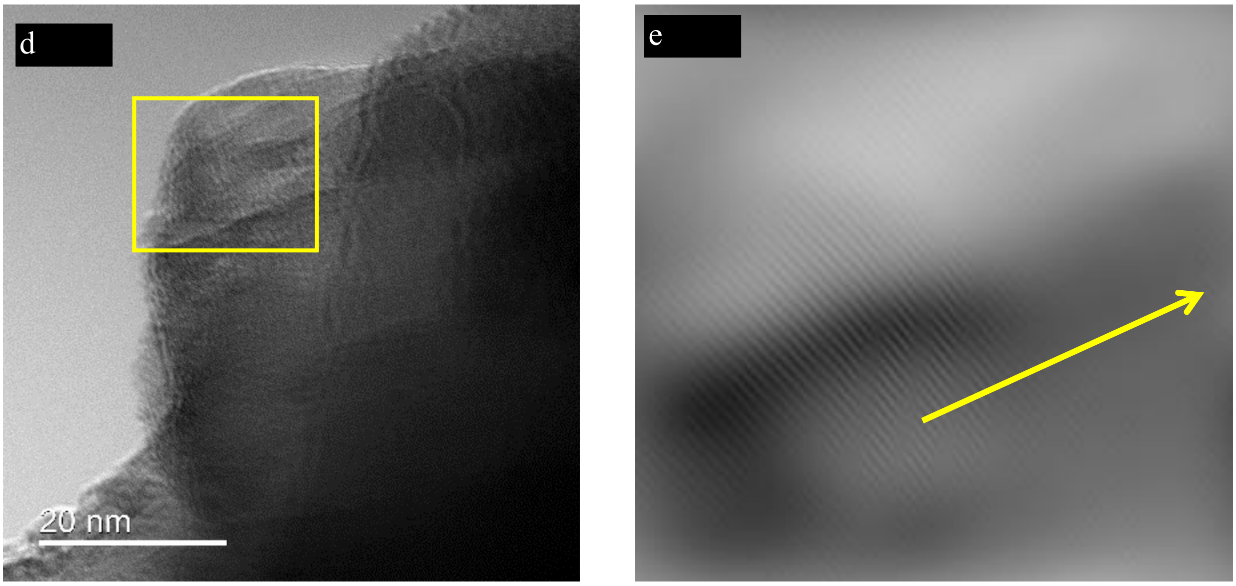

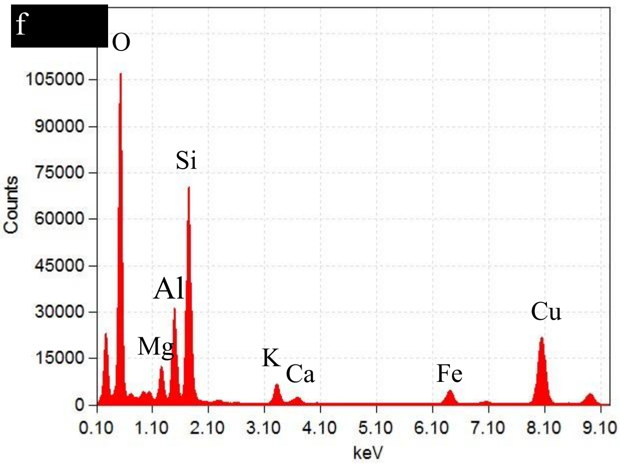

Element		(keV)	Mass%
O K		0.525	40.45
Na K*		1.041	0.78
Mg K*		1.253	4.71
Al K*		1.486	12.93
Si K	(Ref.)	1.739	30.39
S K*		2.307	0.24
K K*		3.312	4.04
Ca K		3.690	1.14
Fe K		6.398	5.33
Total			100.00

Figure S1: a) Low-resolution (LR) TEM image of possible pargasite particle (R050321), b). High-resolution (HR) TEM image of possible pargasite particle, detail 1 c) IFFT of HR TEM image of possible pargasite particle, detail 1 yellow arrow indicates the crystallographic direction of lattice planes of interest, which d_{hkl} is 1.15 Å d). HR TEM image of possible pargasite particle, detail 2, e) IFFT of HR TEM image of possible pargasite particle, detail 2 ; the yellow arrow indicates the crystallographic direction of lattice planes of interest, which d_{hkl} is 1.23 Å and it corresponds to the (0 4 4) plane of pargasite f) standardless quantitative EDXS spectrum of possible pargasite particle.

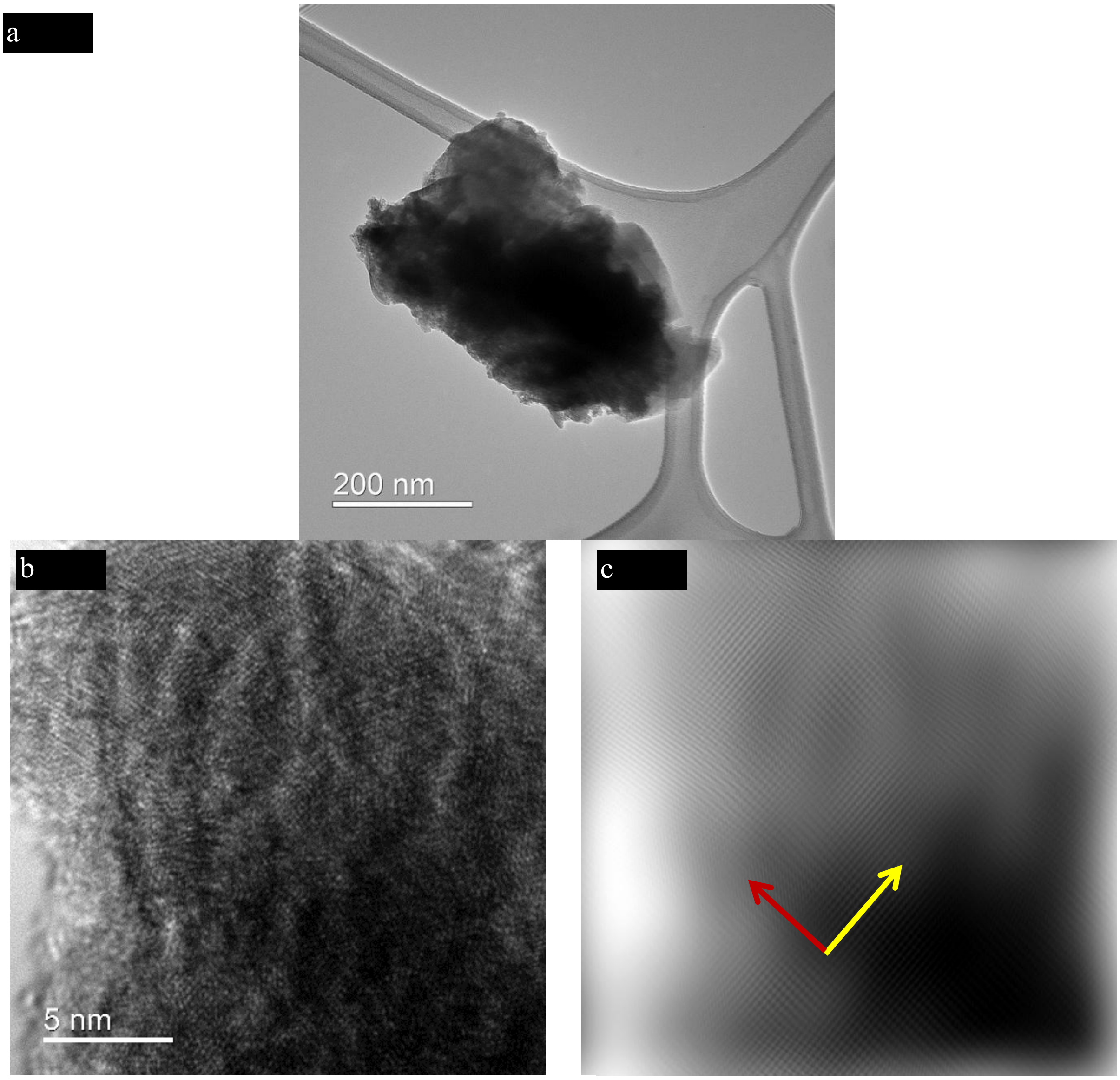

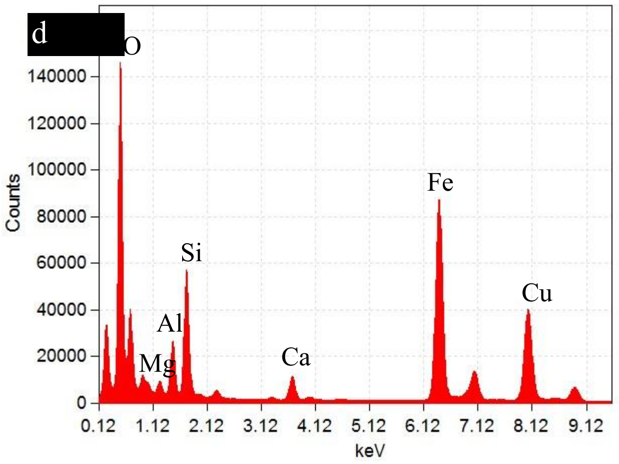

Element		(keV)	Mass%
O K		0.525	24.95
Na K		1.041	0.28
Mg K		1.253	1.17
Al K		1.486	4.87
Si K		1.739	11.29
S K		2.307	0.64
K K		3.312	0.34
Ca K		3.690	3.36
Fe K	(Ref.)	6.398	53.09
Total			100.00

Figure S2: a) LR TEM image of possible aenigmatite particle (R061088), b). HR TEM image of possible aenigmatite particle, c). IFFT of HR TEM image of possible aenigmatite particle; the red arrow indicates the crystallographic direction of lattice planes of interest, which d_{hkl} is 1.75 Å and it corresponds to the (-5 1 3) plane of aenigmatite, and the yellow arrow indicates the crystallographic direction of lattice planes of interest,

which d_{hkl} is 2.3 Å and it corresponds to the (4 -2 1) plane of aenigmatite, d) standardless quantitative EDXS spectrum results of possible aenigmatite particle

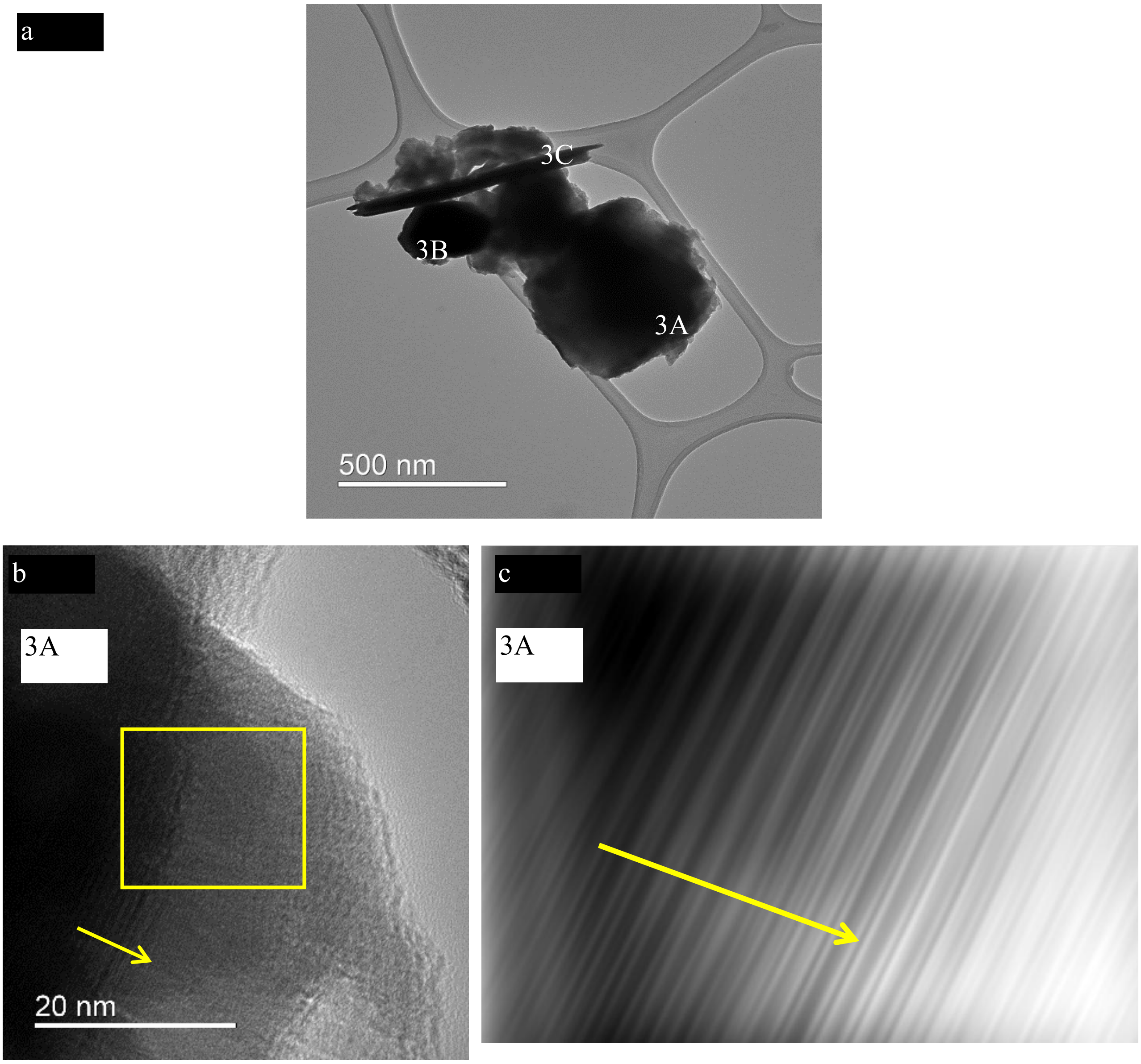

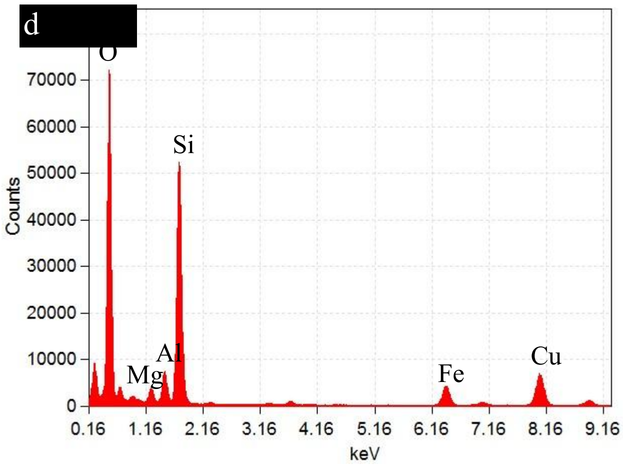

Element		(keV)	Mass%
O K		0.525	45.45
Na K		1.041	0.05
Mg K		1.253	2.18
Al K		1.486	4.75
Si K	(Ref.)	1.739	37.70
S K		2.307	0.20
K K*		3.312	0.39
Ca K		3.690	0.81
Fe K		6.398	8.46
Total			100.00

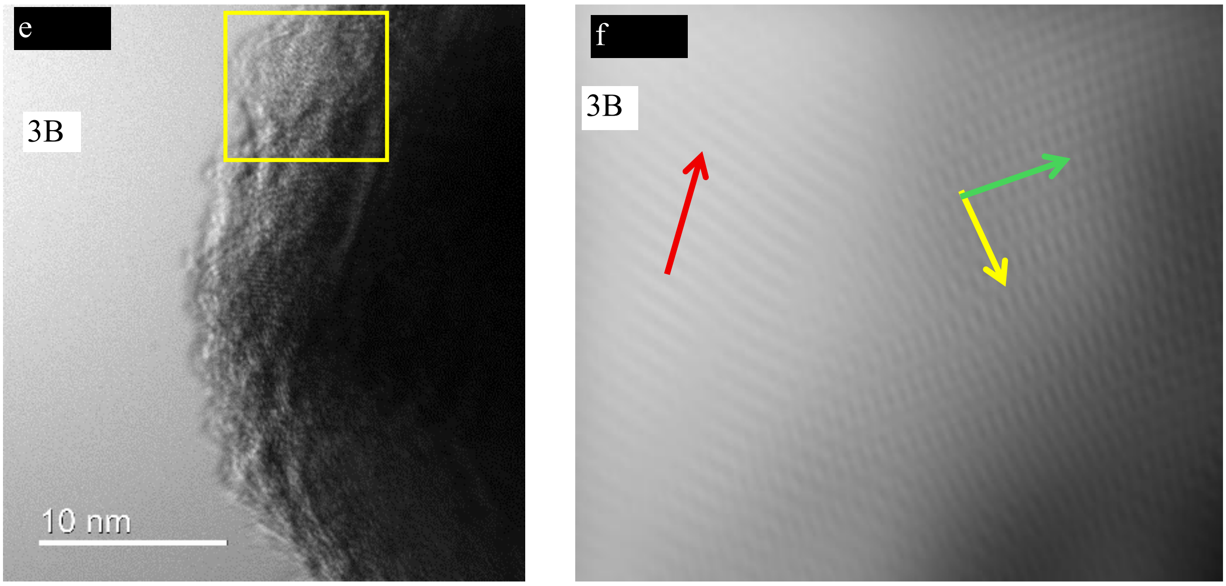

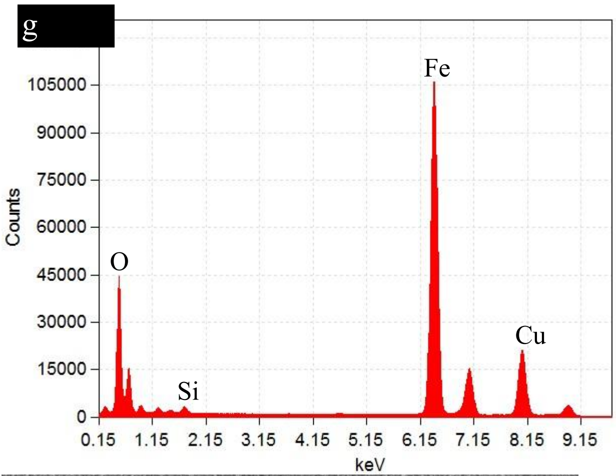

Element		(keV)	Mass%
O K		0.525	10.44
Na K			
Mg K		1.253	0.41
Al K		1.486	0.17
Si K*		1.739	0.55
Fe K	(Ref.)	6.398	88.43
Total			100.00

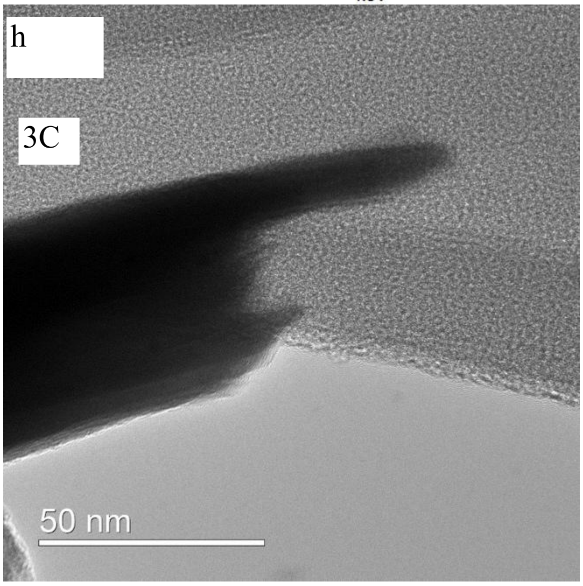

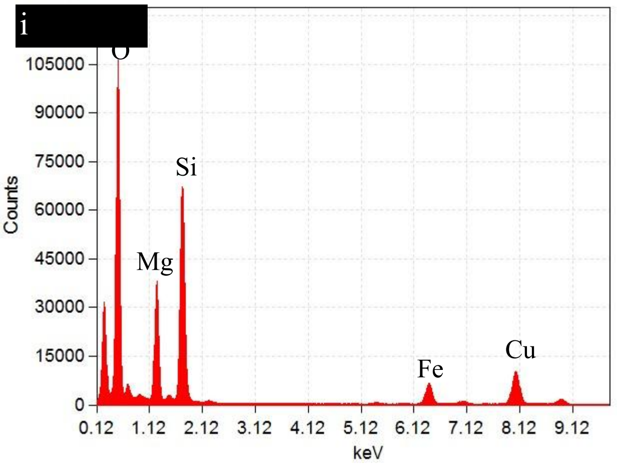

Element		(keV)	Mass%
O K		0.525	40.45
Na K		1.041	0.07
Mg K		1.253	16.81
Al K		1.486	0.84
Si K	(Ref.)	1.739	32.53
S K		2.307	0.03
Cr K*		5.411	0.42
Fe K		6.398	8.85
Total			100.00

Figure S3: a) LR TEM image of aggregate 3, b) HR TEM image of possible augite particle (3A, R061086). The yellow box indicates the IFFT area; the yellow arrow indicates the crystallographic direction of lattice planes of interest, which d_{hkl} is 4.45 Å and it corresponds to the (0 2 0) plane of augite, c) IFFT of HR TEM image of possible augite particle (3A); the yellow arrow indicates the crystallographic direction of lattice planes of interest, which d_{hkl} is 5.98 Å, d) standardless quantitative EDXS spectrum spectrum of High-resolution TEM image of possible augite particle (3A), e). HR TEM image of possible hematite particle (3B, R061086); the red arrow indicates the crystallographic direction of lattice planes of interest, which d_{hkl} is 1.48 Å and it corresponds to the (2 1 4) plane of hematite, the yellow arrow indicates the crystallographic direction of lattice planes of interest, which d_{hkl} is 3.7 Å and it corresponds to the (0 1 2) plane of hematite, and the green arrow indicates the crystallographic direction of lattice planes of interest, which d_{hkl} is 1.16 Å and it corresponds to the (0 1 8) plane of hematite, f) IFFT of HR TEM image of possible hematite particle (3B), g) standardless quantitative EDXS spectrum spectrum of possible hematite particle (3B) h). HR TEM image of particle 3C, i) standardless quantitative EDXS spectrum spectrum of particle 3C

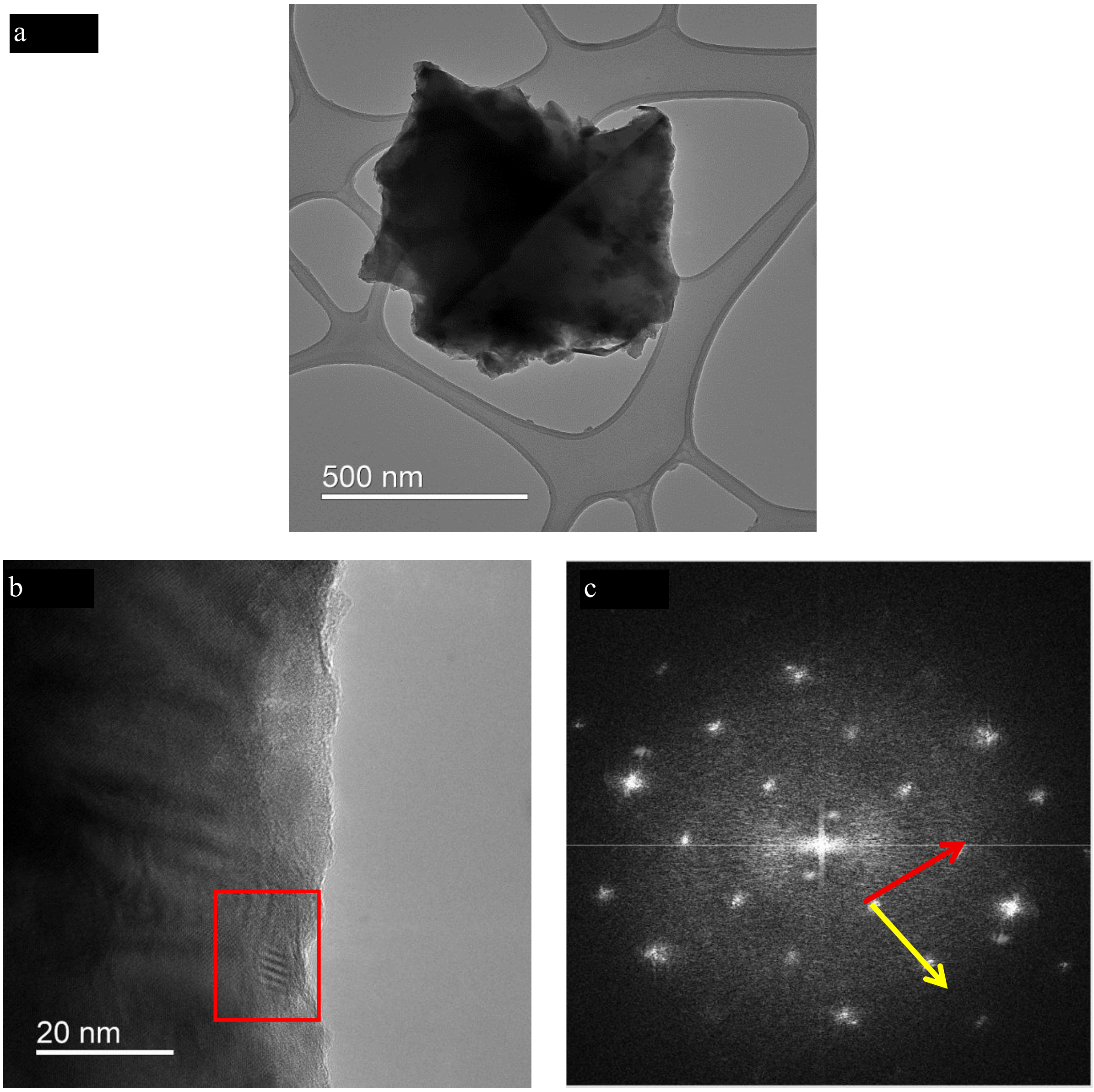

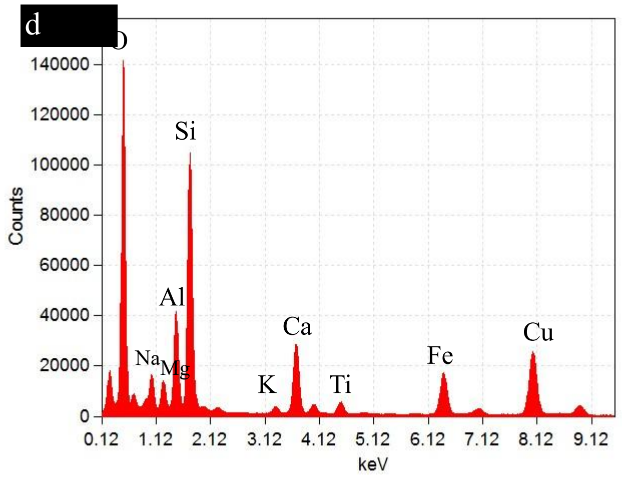

[illegible]

Element		(keV)	Mass%
O K		0.525	30.59
Na K		1.041	3.16
Mg K		1.253	2.90
Al K		1.486	9.97
Si K	(Ref.)	1.739	26.40
P K*		2.013	0.40
S K		2.307	0.12
K K		3.312	1.10
Ca K		3.690	11.04
Ti K*		4.508	2.02
Fe K		6.398	12.31
Total			100.00

Figure S4: a) LR TEM image of particle 4 (MGS-1), b) HR TEM image of particle 4 (the red box indicates moirée fringes effect), c) FFT of HR TEM image of particle 4; the red arrow indicates the crystallographic direction of lattice planes of interest, which d_{hkl} is 3.99 Å, and the yellow arrow indicates the crystallographic direction of lattice planes of interest,

which d_{hkl} is 4.8 Å, e) standardless quantitative EDXS spectrum of particle 4

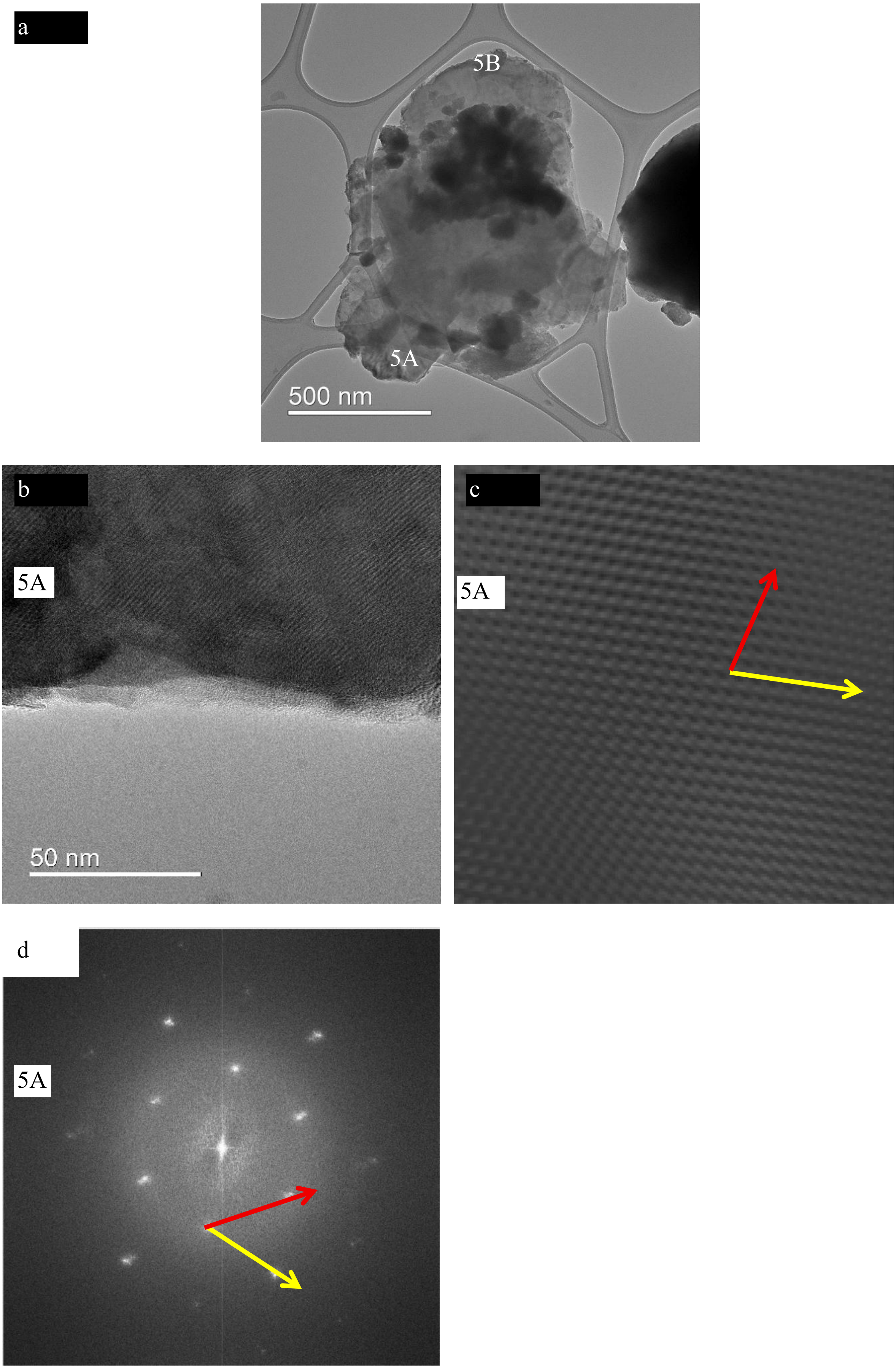
a
5B
5A
500 nm
b
5A
50 nm
c
5A
d
5A

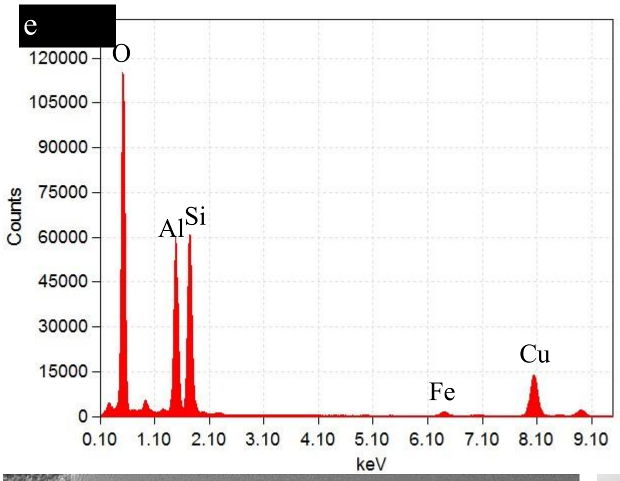

Element		(keV)	Mass%
O K		0.525	44.82
Na K			
Mg K		1.253	0.58
Al K		1.486	26.00
Si K	(Ref.)	1.739	26.80
S K		2.307	0.14
Fe K		6.398	1.67
Total			100.00

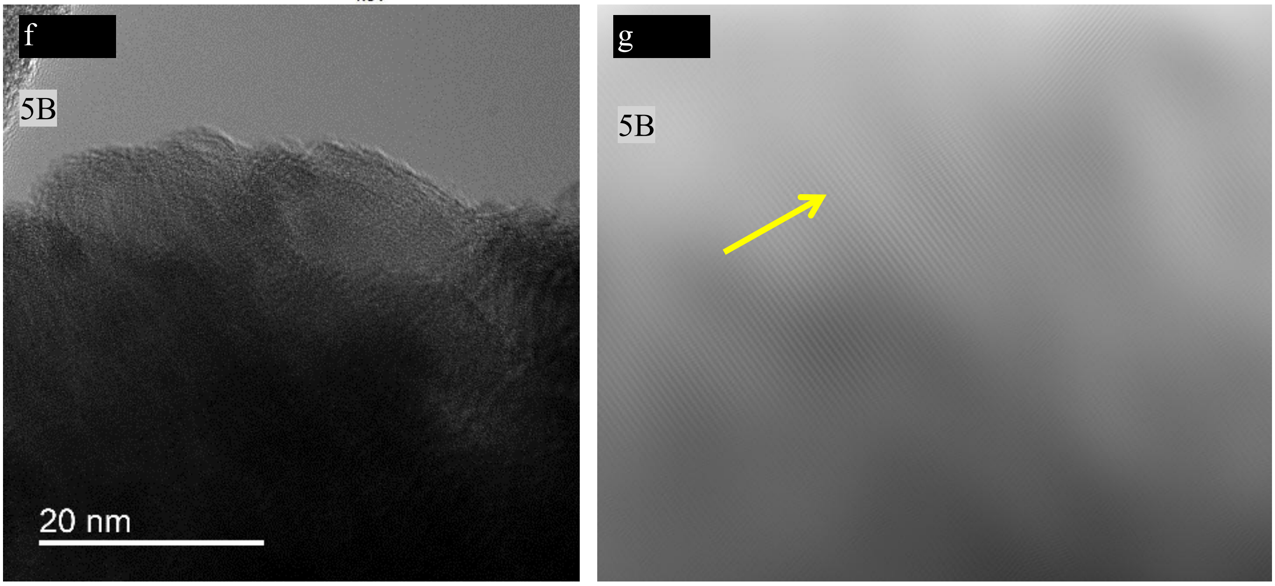

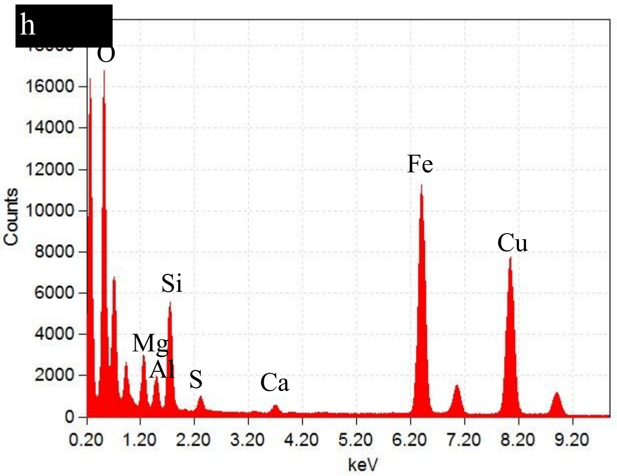

Element		(keV)	Mass%
O K		0.525	25.07
Na K*			
Mg K		1.253	4.38
Al K		1.486	2.69
Si K		1.739	9.21
S K		2.307	1.70
K K		3.312	0.24
Ca K		3.690	1.10
Fe K	(Ref.)	6.398	55.61 1
Total			100.00

Figure S5: a) LR TEM image of aggregate 5 (MGS-1), b) HRTEM image of particle 5A, c) IFFT of HR TEM image of particle 5A; the - red arrow indicates the crystallographic direction of lattice planes of interest, which d_{hkl} is 3.19 Å, and the yellow arrow indicates the crystallographic direction of lattice planes of interest, which d_{hkl} is 3.15 Å, d) FFT of HR TEM image of particle 5A; the red arrow indicates the crystallographic direction of lattice planes of interest, which d_{hkl} of 3.09 Å, and the yellow arrow indicates the crystallographic direction of lattice planes of interest, which d_{hkl} is 3.33 Å, e) standardless quantitative EDXS spectrum of

particle 5A, f) HR TEM image of 5B particle, g) IFFT of HR TEM image of particle 5B; the yellow arrow indicates the crystallographic direction of lattice planes of interest, which d_{hkl} of 1.51 Å h) standardless quantitative EDXS spectrum of particle 5B

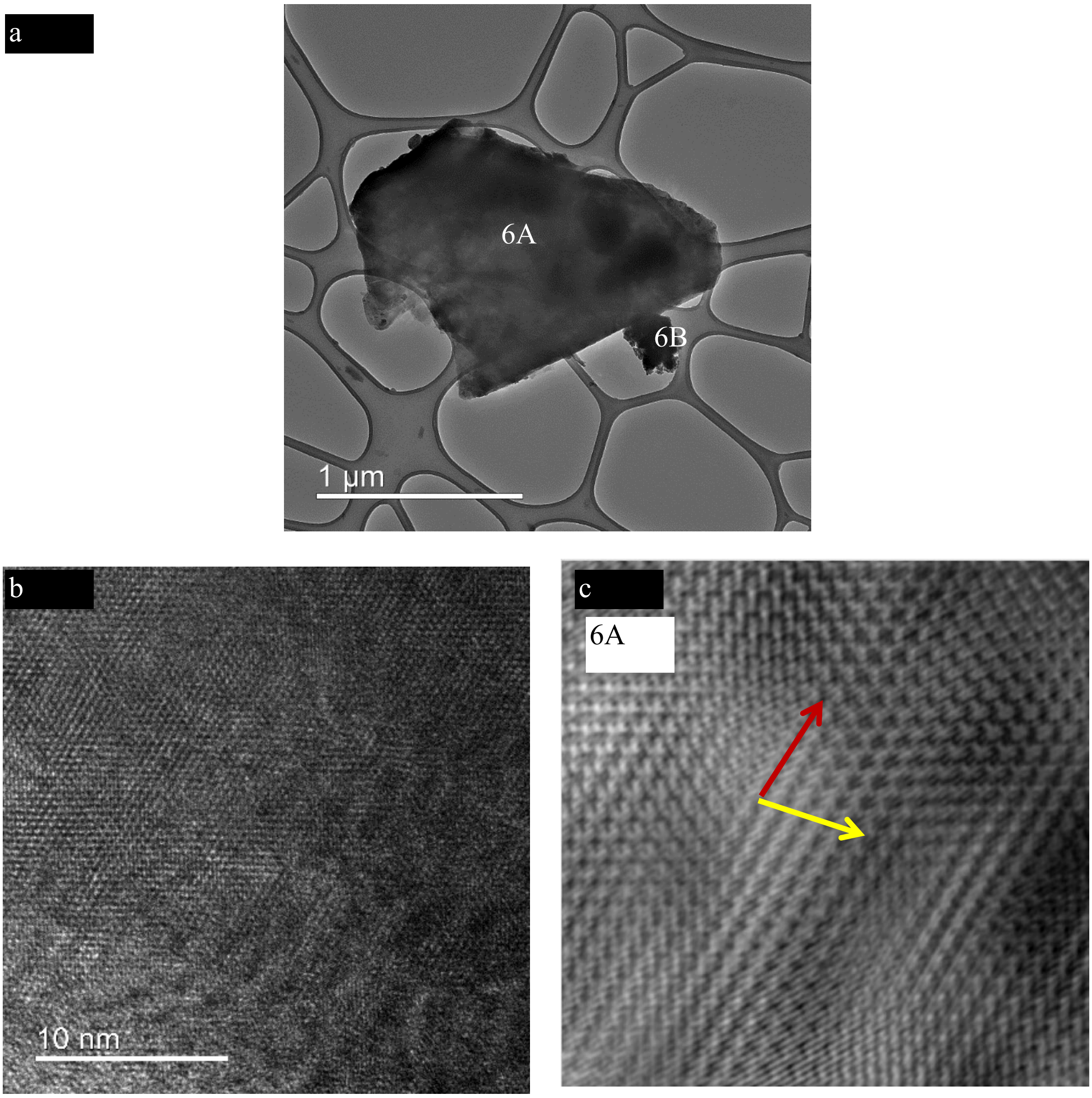

d

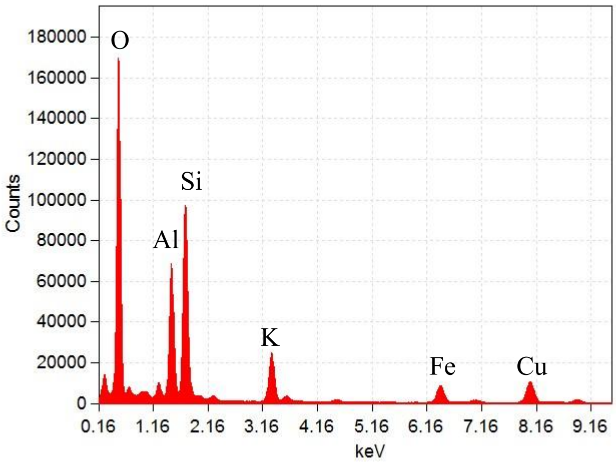

Element	(keV)	Mass%
O K	0.525	38.52
Na K	1.041	0.40
Mg K	1.253	2.03
Al K	1.486	17.37
Si K (Ref.)	1.739	25.05
P K*	2.013	0.39
S K		
K K	3.312	9.31
Ca K		
Ti K*	4.508	0.53
Fe K	6.398	6.40
Total		100.00

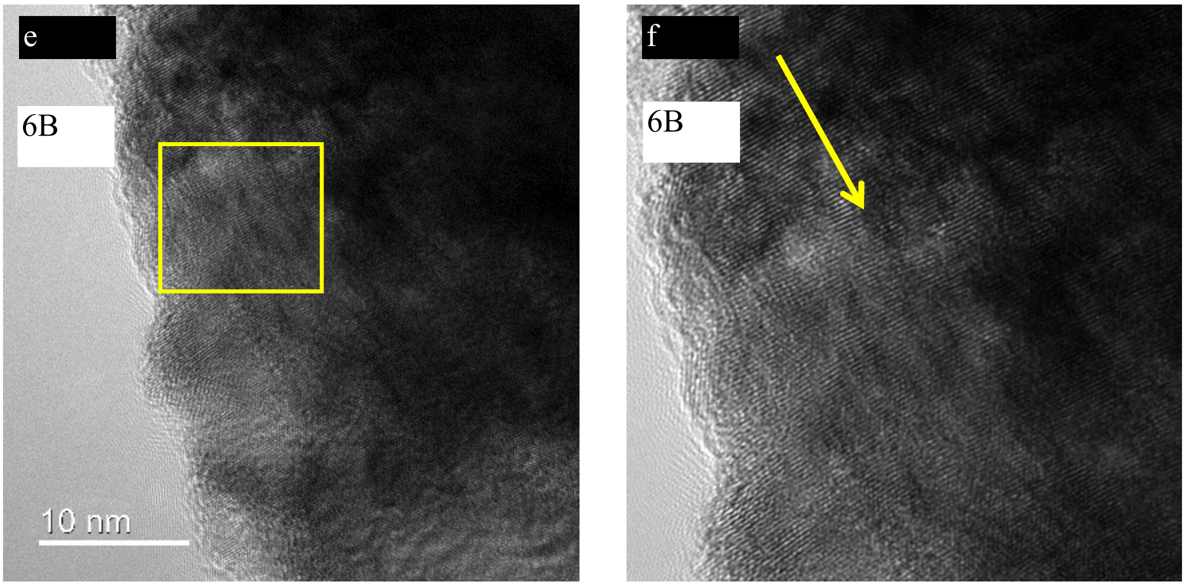

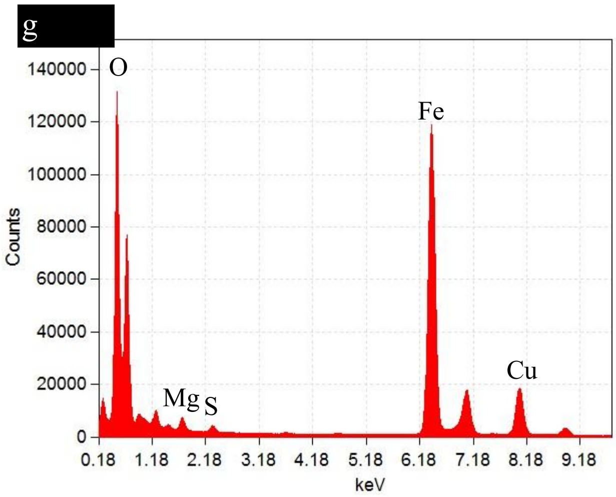

Element	(keV)	Mass%
O K	0.525	22.54
Na K	1.041	0.11
Mg K	1.253	1.11
Al K	1.486	0.29
Si K	1.739	0.99
S K	2.307	0.67
Ca K*	3.690	0.17
Fe K* (Ref.)	6.398	74.13
Total		100.00

Figure S6: a) LR TEM image of aggregate 6, b) HR TEM image of particle 6A, c) IFFT of HR TEM image of particle 6A; the red arrow indicates the crystallographic direction of lattice planes of interest, which d_{hkl} of 3.43 Å, and the yellow arrow indicates the crystallographic direction of lattice planes of interest, which d_{hkl} is 3.53 Å, d) standardless quantitative EDXS spectrum of particle 6A, e) HR TEM image of possible fayalite particle (6B). The yellow box indicates a further zoom-in, f) HR TEM image (related to the yellow ROI area) of possible fayalite particle (6B, R070157); the yellow arrow indicates the crystallographic direction of lattice planes of interest, which d_{hkl} is 1.65 Å and it corresponds to the (1 3 3) plane of fayalite, g) standardless quantitative EDXS spectrum of possible fayalite particle (6B).

Supplementary Figure S7. Petri plates containing maize grown in GMF and hMF conditions

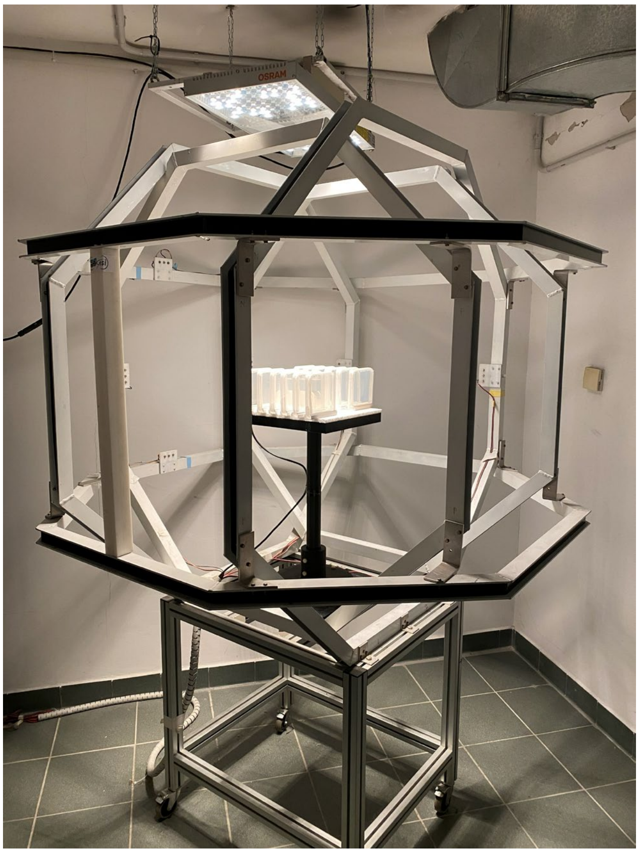

Plants growing inside the Helmholtz coils

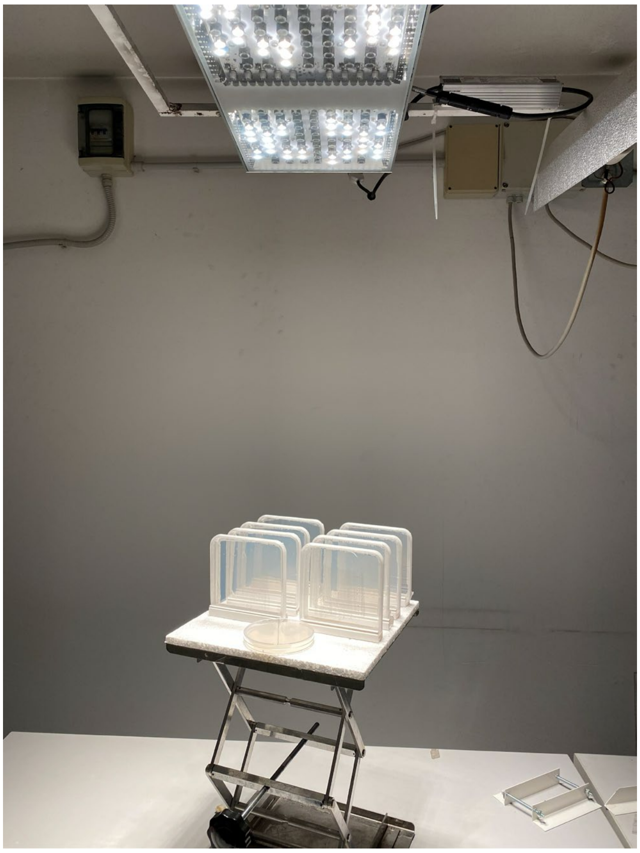

Control (GMF) plants

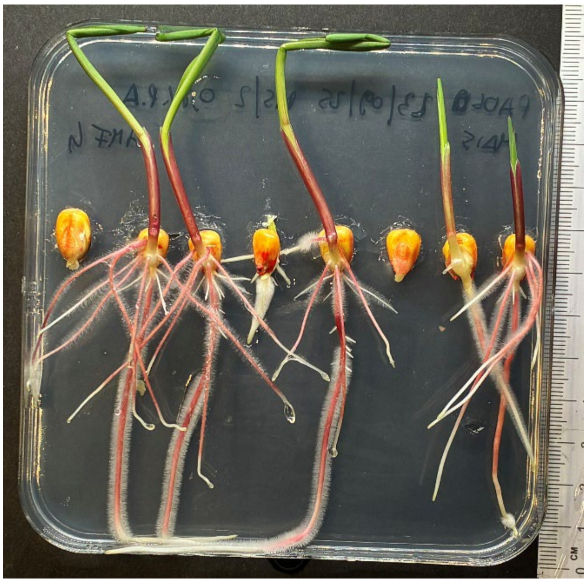

hMF plants

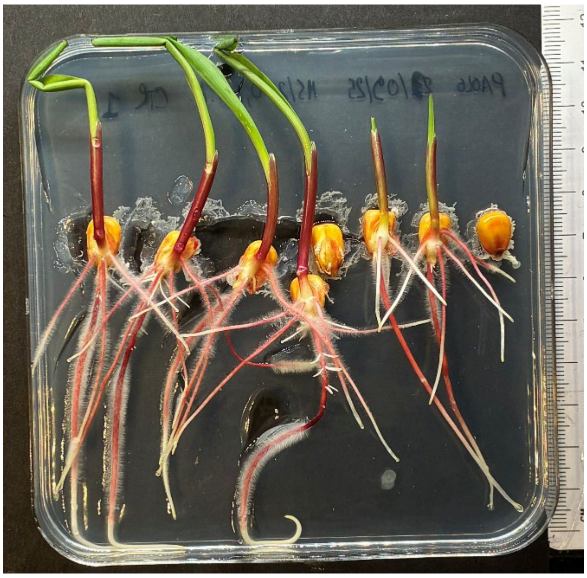

GMF plants

Supplementary Figure S8. Petri plates containing maize grown in GMF and hMF conditions in the presence of MGS-1

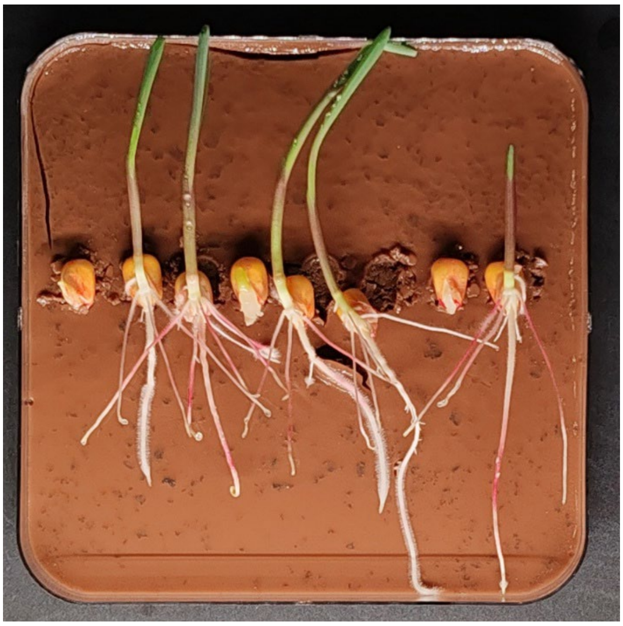

GMF plants

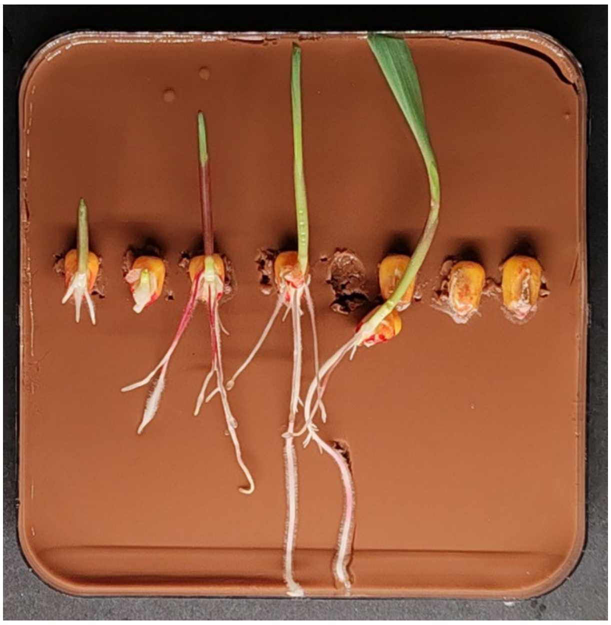

hMF plants

Supplementary Figure S9. Petri plates containing maize grown in 1G and RPM (set to 0.38 G) conditions

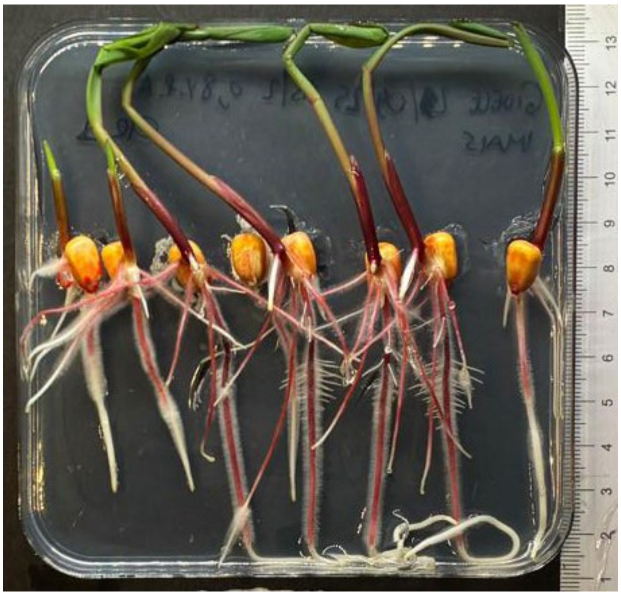

1G plants

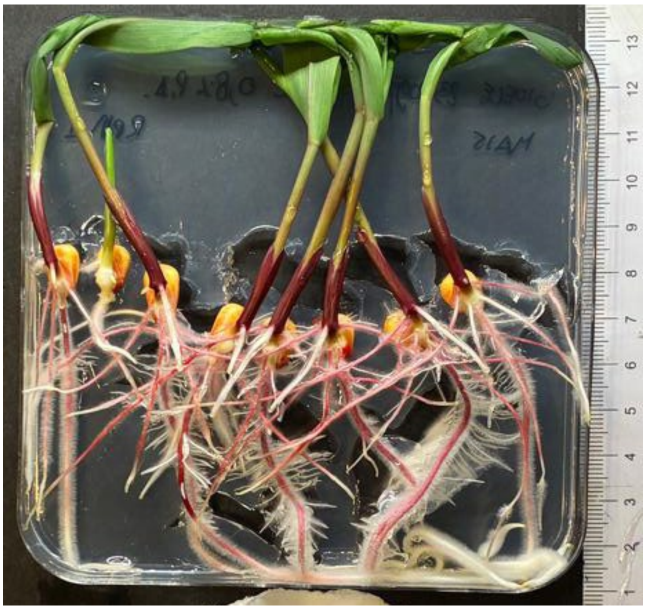

RPM plants

Supplementary Figure S10. Petri plates containing maize grown in 1G and RPM (set to 0.38 G) conditions in the presence of 30% MGS-1

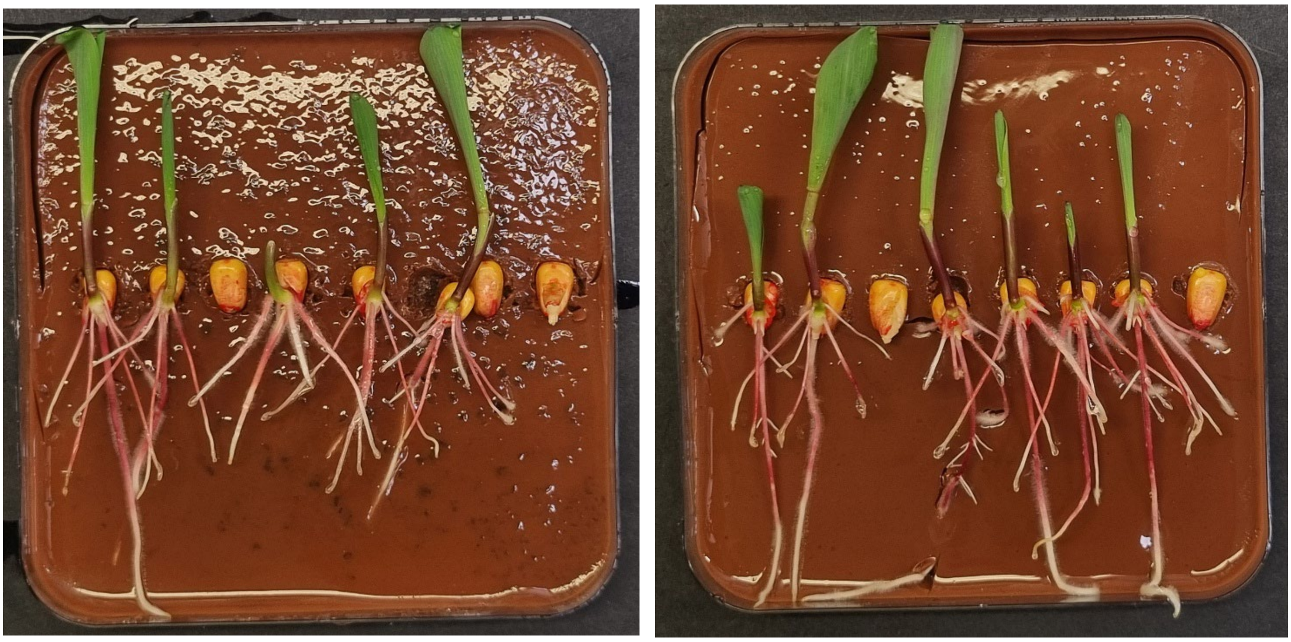

1G plants RPM plants

SUPPLEMENTARY TABLE S1
Safety data sheet
MGS-1 Mars Global Simulant
Version 2.0
Effective date: 2023-11-16

SECTION 1. IDENTIFICATION OF THE SUBSTANCE/MIXTURE AND OF THE COMPANY / UNDERTAKING

1.1 Product identifier

Trade name: MGS-1 Mars Global Simulant

1.2 Relevant identified uses of the substance or mixture and uses advised against:

Product use: Regolith/planetary simulant

1.3 Details of the Supplier of the safety data sheet:

Company: Space Resource Technologies
532 S Econ Cir, Suite 100
Oviedo, FL 32765

Contact: info@spaceresourcetech.com

1.4 Emergency telephone number:
1-800-535-5053 Or contact your regional Poison Control
.

SECTION 2. HAZARDS IDENTIFICATION

2.1 Classification of the substance or mixture according to CLP no. 1272/2008.

**Physical**	**Health**	
Not hazardous	Carcinogen Category 1A<br>Specific Target Organ Toxicity – Repeated exposure	Category 1A (H350i)<br>Category 1 (H372)

2.2 Label elements CLP no. 1272/2008:

Hazard pictograms:

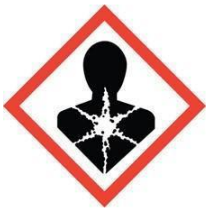

Signal word: Danger

Hazard statements:

H350i May cause cancer by inhalation
H372 Causes damage through prolonged or repeated exposure

Precautionary statements:
Prevention:
Obtain special instructions before use
Do not handle until all safety precautions have been read and understood
Use personal protective equipment as required
Do not breathe dust/fume/gas/mist/vapors/spray
Wash face, hands and any exposed skin thoroughly after handling
Do not eat, drink or smoke when using this product

Response:
If exposed or concerned: Get medical advice/attention

Storage:
Store locked up

Disposal:
Dispose of contents/container to an approved waste disposal plant

Additional labeling:

Carcinogenicity: Natural terrestrial minerals are commonly contaminated with crystalline silica (quartz). Independent laboratory analyses indicate that crystalline silica is present in one or more of the simulant components. The International Agency on Research for Cancer (IARC) has classified silica dust, crystalline as a Group 1, known human carcinogen.

2.3 Other Hazards.
None known.

SECTION 3. COMPOSITION/INFORMATION ON INGREDIENTS

3.2 Mixture:

CAS/EC-no.	REACH-no.	Name	Content %	Classification CLP
1302-54-1	-	Plagioclase	27.1	-
12765-06-9	-	Basaltic Glass	22.9	-
12174-37-7	-	Pyroxene	20.3	-

1317-71-1	-	Olivine	13.7	-
7487-88-9	-	Epsomite	4.0	-
39473-89-7	-	Ferrihydrite	3.5	-
10279-57-9	-	Hydrated Silica	3.0	-
1317-61-9	-	Magnetite	1.9	-
13397-24-5	-	Anhydrite/Gypsum	1.7	-
14476-16-5	-	Siderite	1.4	-
1317-60-8	-	Hematite	0.5	-

3.3 Additional information:
See full text of H-phrases in section 16. Occupational limits are listed in section 8, if these are available.

SECTION 4. FIRST AID MEASURES

4.1 Description of first aid measures:

General information: If any doubt or if symptoms persist, seek medical attention.

Inhalation: Move affected person to fresh air. Get medical attention if any discomfort continues.

Skin contact: Wash exposed skin if irritation exists.

Eye contact: If irritation or discomfort exists, flush eyes lightly with water to remove dust.

Ingestion: Adverse effects not expected from this product.

4.2 Most important symptoms and effects, both acute and delayed:
Inhalation: Irritation may occur.
In case of eye contact: Irritating to eyes.

4.3 Indication of any immediate medical attention and special treatment needed:
Treat symptomatically. If in contact with doctor, bring this safety data sheet with you.

SECTION 5. FIRE FIGHTING MEASURES

5.1 Extinguishing media: Foam, carbon dioxide or dry powder.

Unsuitable extinguishing media:
None known.

5.2 Special hazards arising from the substance or mixture:
The product is not flammable. No special hazards expected.

5.3 Advice for firefighters: No special advice for firefighters.

SECTION 6. ACCIDENTAL RELEASE MEASURES

6.1 Personal precautions, protective equipment and emergency procedures:
Wear appropriate personal protective equipment – see section 8. Avoid contact with eyes. Provide adequate ventilation. Avoid breathing dust. Keep unauthorized and unprotected persons away.

6.2 Environmental precautions: Prevent entry to sewers and public waters.

6.3 Methods and material for containment and cleaning up: Spray lightly with water to avoid creating dust and sweep/shovel into suitable container.

6.4 Reference to other sections: See section 8 and 13 for further information.

SECTION 7. HANDLING AND STORAGE

7.1 Precautions for safe handling: Avoid creating dust. Wear appropriate personal protective equipment – see section 8. Avoid contact with eyes. Avoid breathing dust. Provide adequate ventilation. Keep this product away from food and out of reach of children and pets. Wash hands after handling the product. Remove contaminated clothing. Use only in well ventilated areas.

7.2 Conditions for safe storage, including any incompatibilities: Do not store near heat or open flame. Keep container tightly closed. Keep out of the reach of children. Special containers or storage locations are not required. Incompatible with strong oxidizers.

7.3 Specific end use(s): This product should only be used for applications described in Section 1.2.

SECTION 8. EXPOSURE CONTROLS/PERSONAL PROTECTION

8.1 Control parameters
Occupational exposure limits EH40 (Great Britain):

8.2 Exposure controls

Appropriate technical measures: Airborne concentrations must be kept as low as possible. Provide sufficient ventilation.

CAS-no:	Name:	Limits:
1302-54-1	Plagioclase	No exposure limit.
12765-06-9	Basaltic Glass	No exposure limit.
12174-37-7	Pyroxene	No exposure limit.
1317-71-1	Olivine	No exposure limit
7487-88-9	Epsomite	No exposure limit
39473-89-7	Ferrihydrite	No exposure limit
10279-57-9	Hydrated Silica	2.4 mg/m^3
1317-61-9	Magnetite	No exposure limit
13397-24-5	Anhydrite/Gypsum	4.0 mg/m^3
14476-16-5	Siderite	No exposure limit
1317-60-8	Hematite	No exposure limit
14808–60–7	Crystalline Silica	0.1 mg/m^3

General information/ Hygiene Measures: Wash hands after use

Personal protective equipment: Only CE-marked personal protection equipment should be used.

Respiratory protection: Not required if dust levels are maintained below occupational exposure limits (TLV-TWA of 0.1 mg/m3). For levels above the occupational exposure limits wear an appropriate NIOSH approved respirator.

Hand protection: None required. For hygiene purposes, chemically compatible gloves are appropriate.

Eye protection: Safety glasses should be worn.

Body protection: None required. Confine work clothing to workplace and wash daily.

Measures to avoid environmental exposure: Avoid discharge to lakes, streams, sewers, etc.

SECTION 9. PHYSICAL AND CHEMICAL PROPERTIES

9.1 Information on basic physical and chemical properties:

Appearance: Red powder.
Upper/lower flammability or explosive limits: N/D
Odor: None.
Vapor pressure: N/D
Odor threshold: N/D
Vapor density: N/D
pH: N/D
Relative density: N/D
Melting point/freezing point: N/D
Solubility: Insoluble in water.
Initial boiling point and boiling range: N/D
Flash point: N/D
Evaporation rate: N/D
Flammability: N/D
Partition coefficient (n-octanol/water): N/D
Auto-ignition temperature: N/D
Decomposition temperature: N/D
Viscosity: N/D

9.2 Other information:
-

SECTION 10. STABILITY AND REACTIVITY

10.1 Reactivity: None under normal conditions.

10.2 Chemical stability: Stable under normal storage conditions and recommended use.

10.3 Possibility of hazardous reactions: None known.

10.4 Conditions to avoid: Heat, open flames, sparks.

10.5 Incompatible materials: Strong oxidizers.

10.6 Hazardous decomposition products:
Releases carbon monoxide, carbon dioxide, sulfur monoxide, sulfur dioxide and methane upon combustion.

SECTION 11. TOXICOLOGICAL INFORMATION

11.1 Information on Toxicological effects:

**Acute toxicity:**	Not classified.
**Skin corrosion/irritation:**	Not classified.
**Serious eye damage/irritation:**	Not classified.
**Respiratory or skin sensitisation:**	Not classified.
**Germ cell mutagenicity:**	Not classified.
**Carcinogenicity:**	The International Agency on Research for Cancer (IARC) has classified silica dust, crystalline as a Group 1, known human carcinogen.
**Reproductive toxicity:**	Not classified.
**Specific target organ toxicity - single exposure:**	May cause respiratory irritation.
**Specific target organ toxicity – repeated exposure:**	Adverse health effects including silicosis, lung cancer, autoimmune and chronic kidney diseases, tuberculosis, and non-malignant respiratory diseases are attributed to respirable crystalline silica.
**Aspiration hazard:**	Not classified.
**Additional information:**	Inhalation: Irritation may occur.<br>In case of eye contact: Irritating to eyes.

SECTION 12. ECOLOGICAL INFORMATION

12.1 Toxicity: Not classified.

12.2 Persistence and degradability:
The product is biodegradable.

12.3 Bioaccumulative potential: No data.

12.4 Mobility in soil: No data.

12.5 Result of PBT and vPvB assesment:
No CPSR are created.

12.6 Other adverse effects: None known.

SECTION 13. DISPOSAL CONSIDERATIONS

13.1 Waste treatment methods: Must be disposed of in accordance with local and national regulations. The coding of a waste stream is based on the application of the product by the consumer.

Contaminated packing: Packaging which contains leftovers from the product must be disposed of in the same way as the product

SECTION 14. TRANSPORT INFORMATION

This product is not classified as dangerous to transport.

	**ADR/RID**	**IMDG/IMO**
**14.1 UN number**	-	-
**14.2 UN proper shipping name**	-	-
**14.3 Transport hazard class(es)**	-	-
**14.4 Packing group**	-	-

**14.5 Environmental hazards - MP**	No<br>-	No<br>-
**Other informations**	LQ: -<br>TUNNEL: -	LQ: -<br>TUNNEL: -

14.6 Special precautions for user:
-

14.7 Transport in bulk according to Annex II of MARPOL 73/78 and the IBC Code:
-

SECTION 15. REGULATORY INFORMATION

15.1 Safety, health and environmental regulations/legislation specific for the substance or mixture:

Sources:
Pressure Equipment (Amendment) Regulations 2011. Chemicals (Hazard Information and Packaging for Supply) Regulations 2009. Control of Substances Hazardous to Health Regulations 2002 (as amended).

Merchant Shipping (Dangerous Goods and Marine Pollutants) Regulations 1997. Reporting of Injuries, Diseases and Dangerous Occurrences Regulations 1995 (as amended). Personal Protective Equipment

Regulations 2002. Personal Protective Equipment at Work Regulations 1992. Hazardous Waste (England and Wales) Regulations 2005(as amended). EC regulation 1907/2006 (REACH) Directive 2000/532/EC. Seveso directive: 96/82/EC. EU 830/2015. CLP 1272/2008.

Additional information: -

15.2 Chemical safety assessment:
Chemical safety assessments have not been performed for this product.

SECTION 16. OTHER INFORMATION

Full text of H-phrases as mentioned in section 3:
H350i May cause cancer by inhalation
H372 Causes damage through prolonged or repeated exposure

Additional information:
This information is based on our current knowledge and is intended to describe the product for the purposes of health, safety and environmental requirements only. It should not therefore be construed as guaranteeing any specific property of the product.

SDS Prepared on: 3/31/2021
Last Known Revision: 11/16/2023

Supplementary Table S2. Primers used in this work

Gene code	Gene locus	Decription	Forward	Reverse
Elfa2	LOC100037748	Elongation factor alpha 2	GCTGAGCGTGAGAGAGGTAT	TGATGAAGTCGGTGTCCA
ROBH1	LOC778438	Respiratori Burst Oxidase Homolog 1	ACTCTCGCATAGGTAAGCCAC	TTGTTTTGTGAGCGTCGGAG
SOD1	LOC100282741	Superoxide Dismutase 1	TTCACTTCTTCGAGGACCCC	CATTGGTAGTGTCGCCGAAG
CAT1	LOC542369	Catalase 1	ACCCATGAACACCGTACCAT	TCGATCTTACATGCTCGGCT
APX1	LOC103639279	L-Ascobrate Peroxidase 1, cytosolic	CCGCCCTCTTGTGGAGAAAT	CTCATCCTACAGAGCACACGA
GSR1	LOC541986	Glutathione Reductase 1	TTGGAATACACCGGCAGAGT	GAAAATAGCTCGGCGGACAG
ZmPIN1a	LOC103637824	Probable auxin efflux carrier component 1c	GCGCAAACTCATCCGTAACC	GGCATCTCGAAGTTCCACCT
ZmPIN1b	LOC103627718	Auxin efflux carrier component 1a	CCACATGTTCGTCTGGAGCT	GTAGTCGTCCCTGTCCTTGC
ZmPIN1c	LOC103654258	Auxin efflux carrier component 1a	ATCCTGATCATGGTGTGGCG	ATGGAGTGCAGGACGATGTC
ISCA1	LOC100282007	Iron-sulfur assembly protein IscA-like 1, mitochondrial	TTTTGCCCCACTGTCCATCA	GACCCTTTGCTGCCTGTAGT
ISCA2	LOC100282014	Iron-sulfur assembly protein IscA-like 2, mitochondrial	ACCAGATACCGAAGCCGTAG	GTAAACGGTGGCGTGATGAG
